# Chemistry and Biological Activity of *Ramalina* Lichenized Fungi

**DOI:** 10.3390/molecules20058952

**Published:** 2015-05-19

**Authors:** Antônio Sérgio Nascimento Moreira, Raimundo Braz-Filho, Vicente Mussi-Dias, Ivo José Curcino Vieira

**Affiliations:** 1 Laboratório de Ciências Químicas, Universidade Estadual do Norte Fluminense Darcy Ribeiro, UENF, Avenida Alberto Lamego 2000, Campos dos Goytacazes, 28013-602 Rio de Janeiro, Brazil; E-Mails: braz@uenf.br (R.B.-F.); curcino@uenf.br (I.J.C.V.); 2 Instituto Federal Fluminense, IFF, Avenida Souza Mota 350, Parque Fundão, Campos dos Goytacazes, 28060-010 Rio de Janeiro, Brazil; 3 Laboratório de Entomologia e Fitopatologia, Universidade Estadual do Norte Fluminense Darcy Ribeiro, UENF, Avenida Alberto Lamego 2000, Campos dos Goytacazes, 28013-602 Rio de Janeiro, Brazil; E-Mail: vicmussi@uenf.br

**Keywords:** lichen, *Ramalina*, biological activitiy, usnic acid, antitumoral

## Abstract

Lichens are a form of symbiont between a fungus and an alga or cyanobacterium, which contains a wide variety of organic compounds with certain secondary metabolite classes typical of these organisms. The *Ramalina* genus has approximately 246 species distributed around the World, of which in this review approximately 118 species with published chemical or biological activity studies of extracts or isolated compounds were cited. From the 153 mentioned compounds, only 27 passed were tested for biological activity, being usnic acid the most studied compound and the one showing the best results in almost all *in vitro* tests performed, although other compounds also presented excellent results as antimicrobial, antitumor and anti-inflammatory agents, among others. Extracts of several species also presented significant results in performed biological tests, demonstrating the potential that these organisms have, in particular, the gender *Ramalina*, to produce bioactive molecules that can be used as a model for the production of pharmaceuticals.

## 1. Introduction

Lichenized fungi (syn. Lichens) constitute a form of symbiotic mutualism between a fungus (micobiont) and an alga or cyanobacteria (photobiont) that contains a large number of organic compounds, some of them specific to these organisms [1], and constitute a stable and independent systematic unit belonging to the Kingdom Fungi (phylum Ascomycota-Basidiomycota) [2]. Until 1998, it was estimated that there was 13,500 species (about 600 genera), which corresponds to 20% of fungi known [3]. In 2005 this estimate rose to 18,500 different species around the World. This demonstrates, in addition to the diversity, the great interest that researchers have been increasing in the study of these special organisms [2].

Lichenized fungi are well known for the diversity of secondary metabolites that they produce [1,4]. This diversity of compounds has made the study of their chemistry attractive since the beginning of organic chemistry, from 1830 to the present day [1]. Many of the metabolites are typical of this class of organisms [1,5,6]. More than 800 compounds have been reported, and among the different classes of compounds are ones containing nitrogen, phosphorus and sulfur, polyols, carbohydrates, aliphatic and cycloaliphatic compounds, aromatic compounds, meta- and para-depsides, depsidones, dibenzofurans, diphenylethers, naphtopyrans, biphenyls, diphenylmethanes, nostoclides, xanthones, quinones, naphthoquinones and usnic acid. Esters, terpenes, steroids, terphenylquinones and derivatives of pulvinic acid also occur [1,6]. Currently 246 species at the genus *Ramalina* are described, which are widely distributed worldwide [5,7]. In this review paper about 110 species were cited, for which the chemistry or biological activity of its crude extracts or any of its isolated compounds was studied. The goal of this review paper is to verify which are the main biological activities and which metabolites have been isolated from lichens of the genus *Ramalina*.

## 2. Chemical Constituents

**Figure 1:**
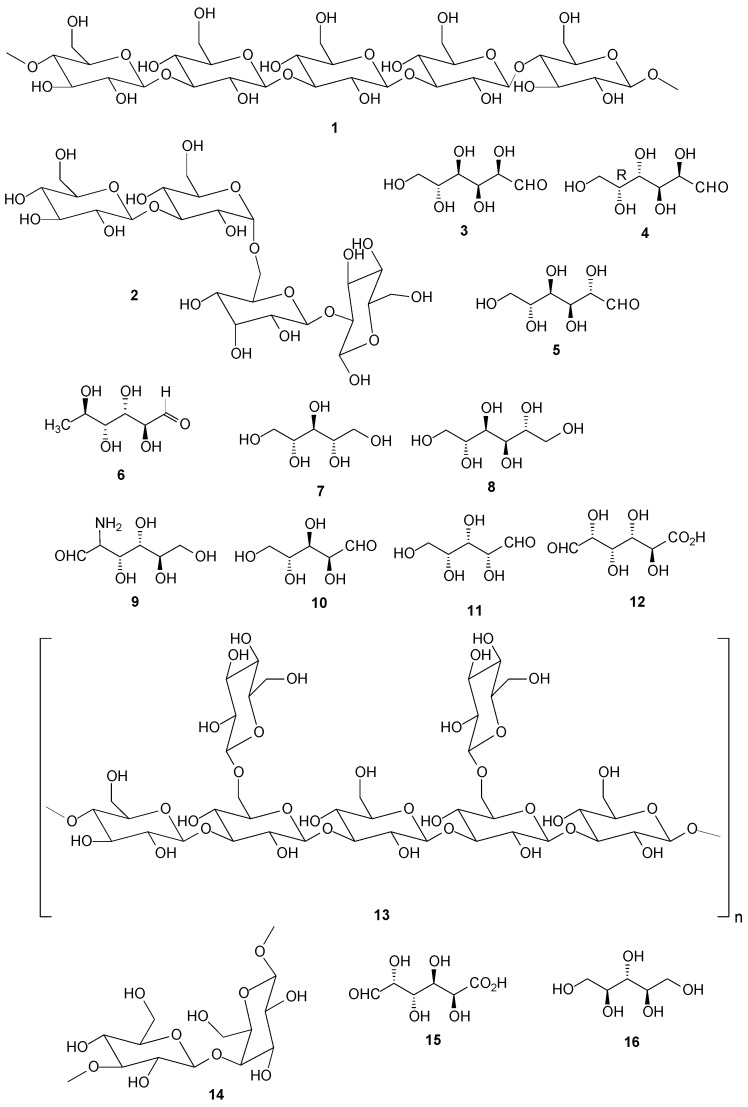
Chemical structures of polysaccharides of *Ramalina* species of lichenized fungi.

**Figure 2:**
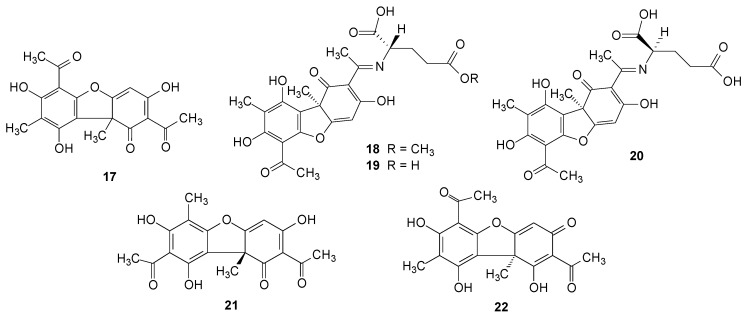
Chemical structures of usnic acid and derivatives from *Ramalina* species of lichenized fungi.

**Figure 3:**
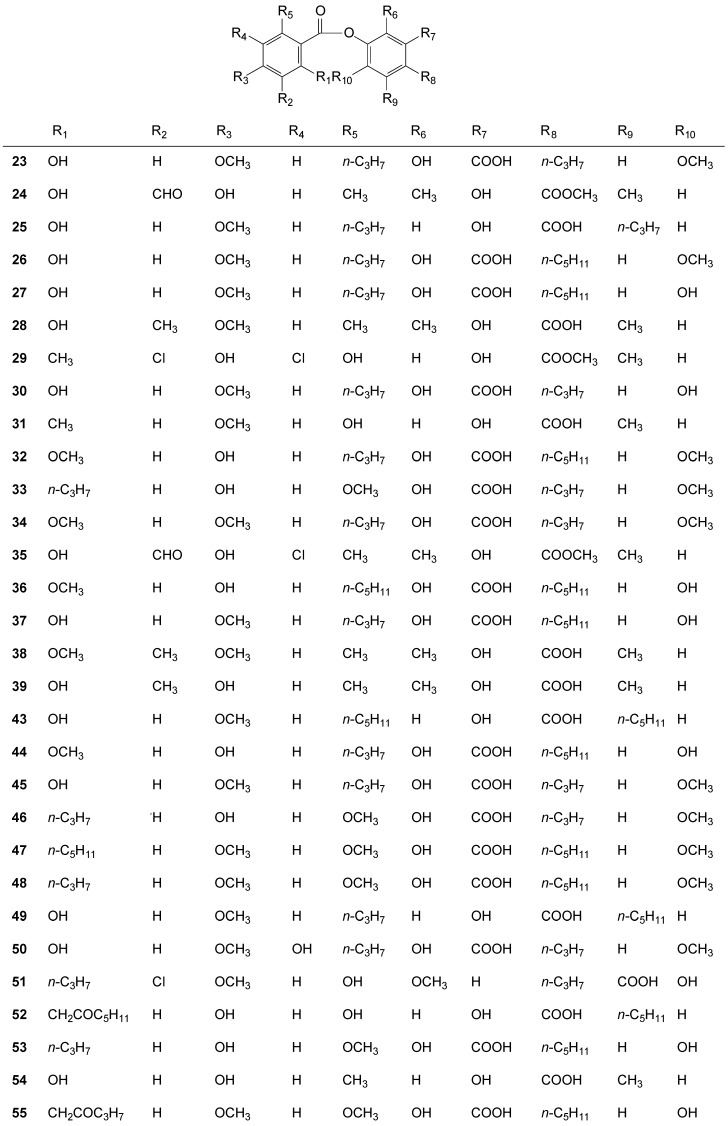

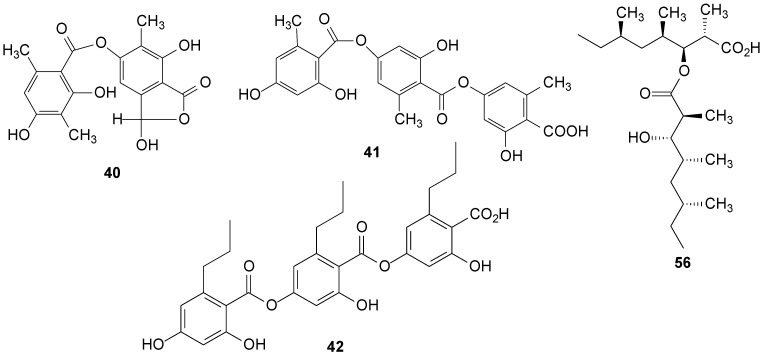
Chemical structures of the depsides and derivatives of the *Ramalina* species of lichenized fungi.

**Figure 4:**
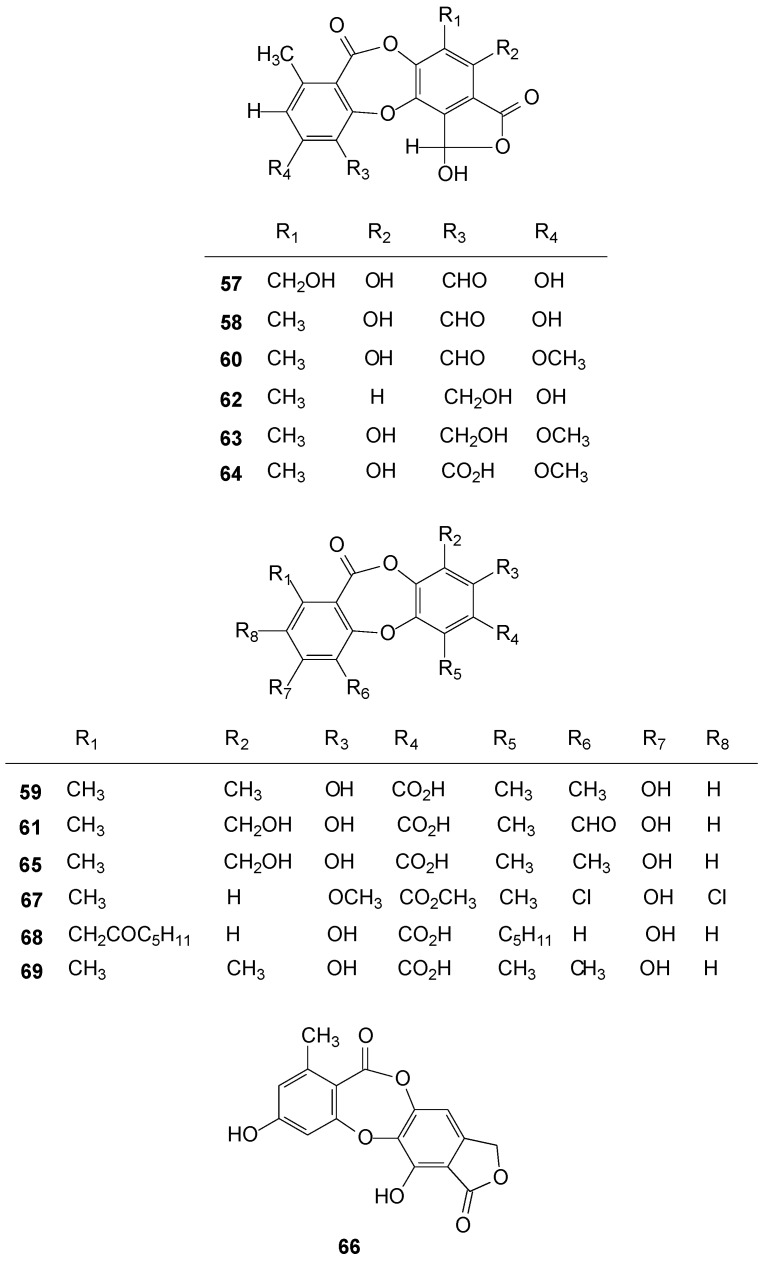
Chemical structures of depsidones from *Ramalina* species of lichenized fungi.

**Figure 5:**
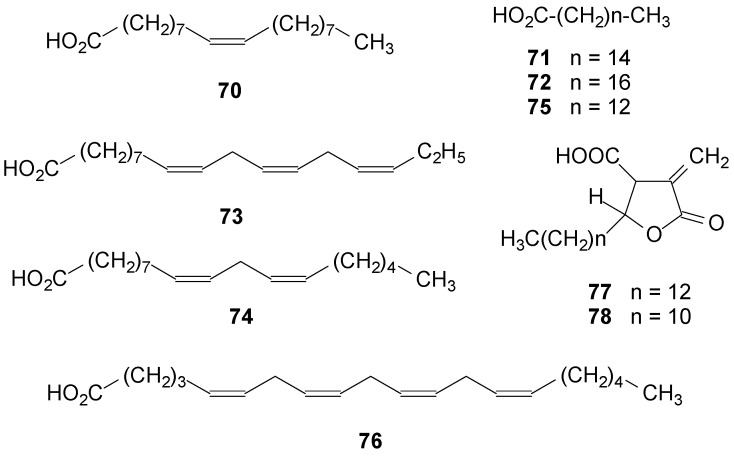
Chemical structures of fatty acids from *Ramalina* species of lichenized fungi.

**Figure 6:**
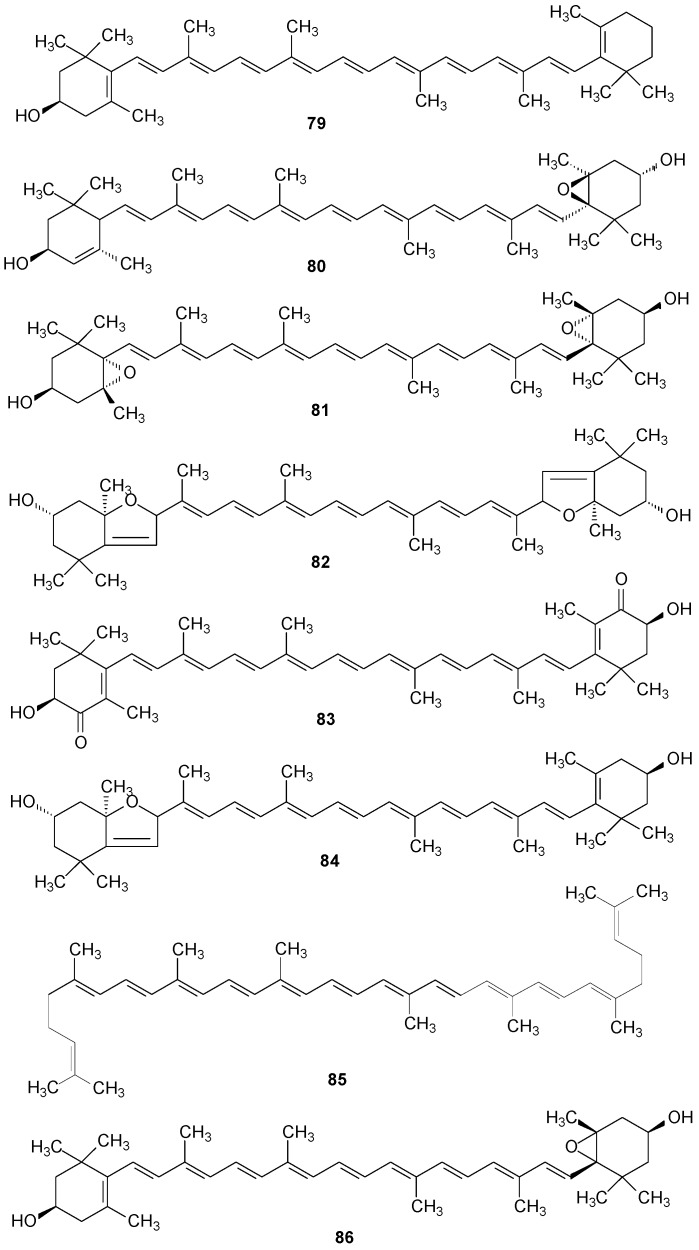

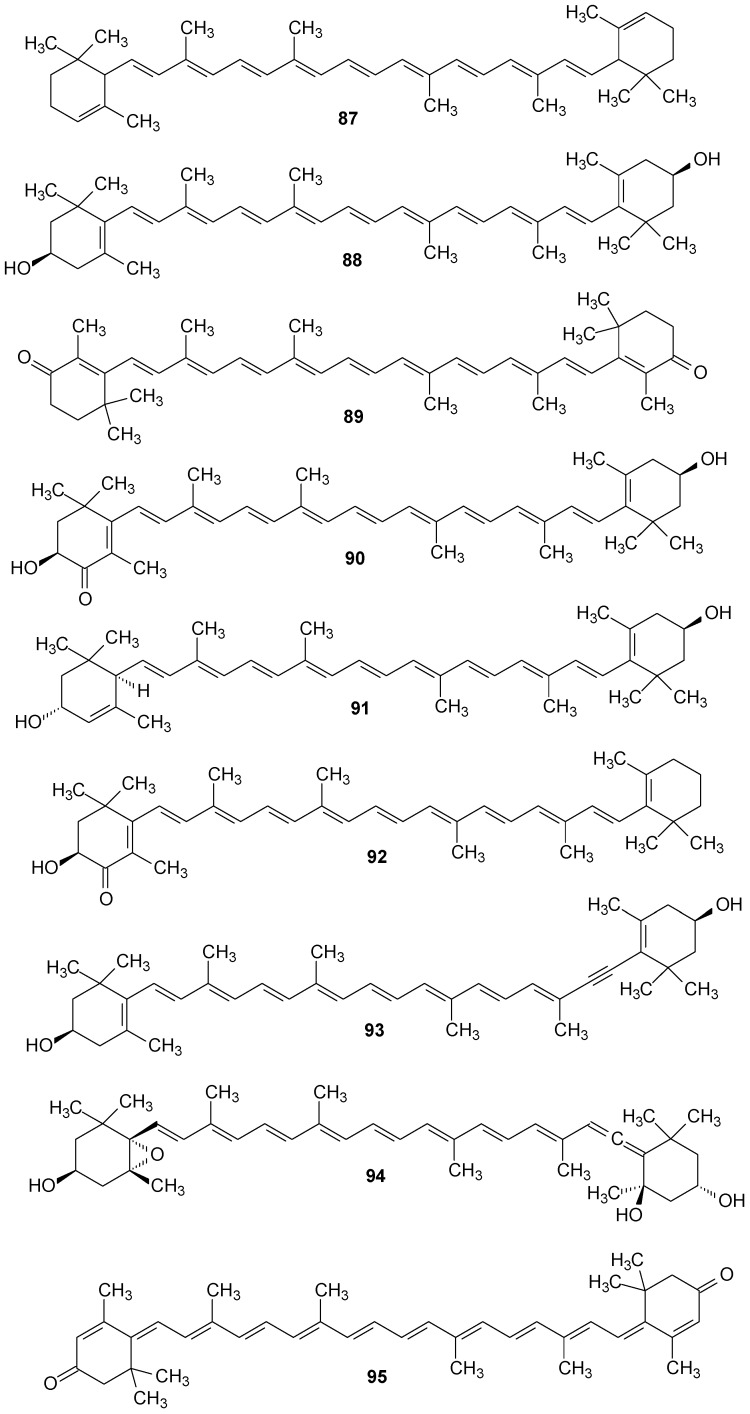
Chemical structures of other compounds: carotenoids of *Ramalina* species of lichenized fungi.

**Figure 7:**
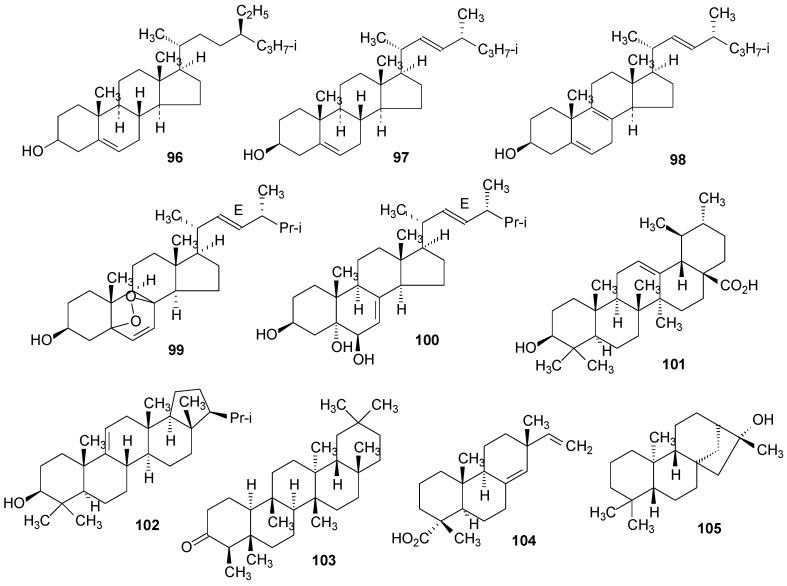
Chemical structures of other compounds: steroids and terpenoids from *Ramalina* species of lichenized fungi.

**Figure 8:**
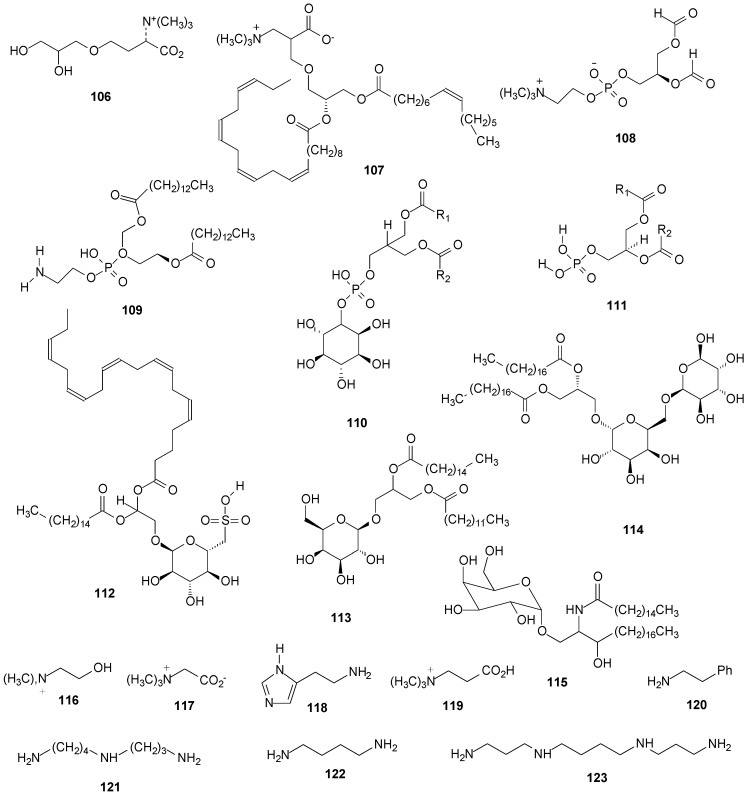
Chemical structures of other compounds: lipids and amines from *Ramalina* species of lichenized fungi.

**Figure 9:**
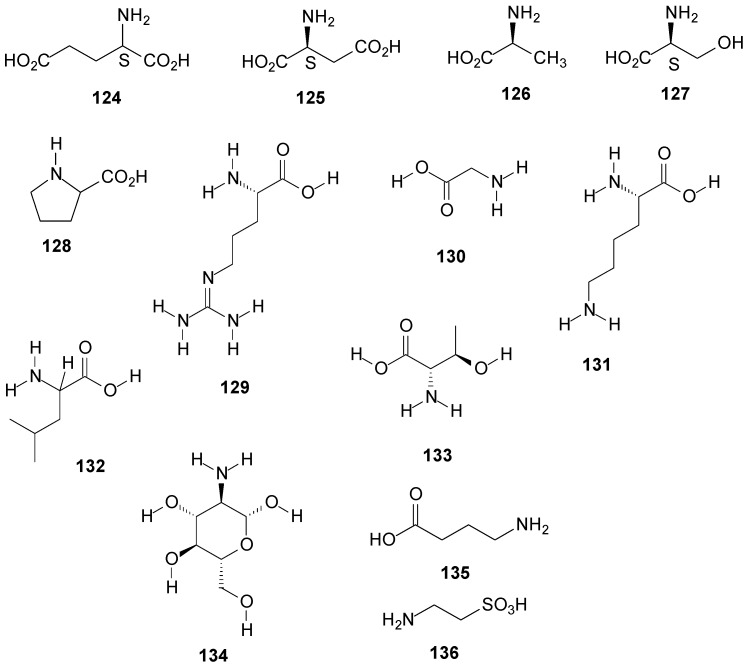
Chemical structures of other compounds: amino acids from *Ramalina* species of the fungi.

**Figure 10:**
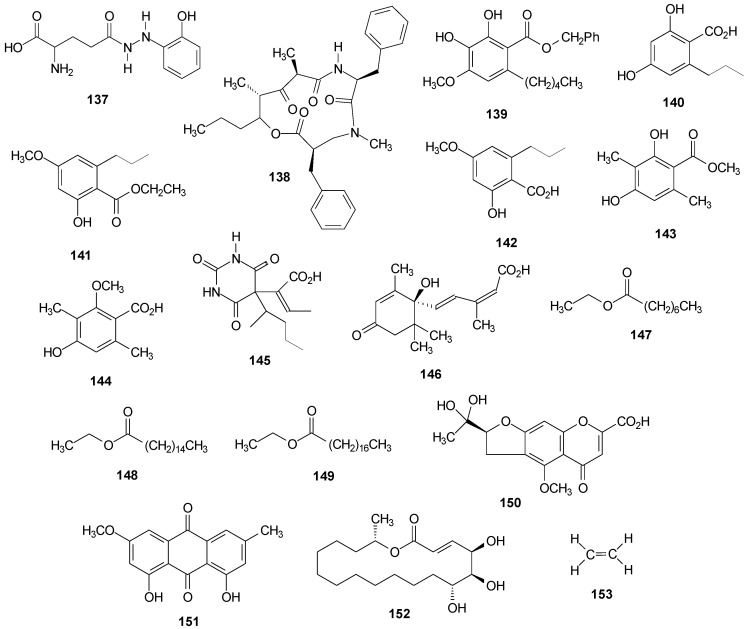
Chemical structures of other compounds from *Ramalina* species of lichenized fungi.

In the researched species of the genus *Ramalina*, a diversity of chemical compounds was found, including both primary and secondary metabolites. Among primary metabolites, carbohydrates were the most abundant ones; amino acids, glycolipids, glycosphingolipids and polyols were also detected. Among secondary metabolites, usnic acid deserves attention because of its frequent mention. Derivatives of this acid (usimines) [8,9,10,11], (+)-*iso*-usnic acid [12] and usninic acid [13] were also found. Depsides, depsidones, fatty acids, sterols and monocyclic aromatic compounds were found among the most frequent ones, besides other classes of compounds at very low frequency. Each compound and its respective origin (lichenized species) are listed in Tables S1–S10 (Supplementary Material), and their structures are represented in Figure 1, Figure 2, Figure 3, Figure 4, Figure 5, Figure 6, Figure 7, Figure 8, Figure 9 and Figure 10.

### 2.1. Carbohydrates

The main polysaccharides of *Ramalina* are linear glucans and heteropolysaccharides. The first group consists of compounds with α and β configurations that possess in their structures (1→3)- and (1→4)- (**1**) bonds in different proportions. The α-configuration corresponds to the the isolichenan class and β to the lichenan one. The second group is heteropolysaccharidic branched-chain-containing galactose and mannose (galactomannan), where the most abundant feature bonds (1→6)- containing α-d-Man*p* (**2**) as the main chain and α- or β-d-Gal*p*, β-d-Gal*f* and α-d-Man*p* as side chains [14,15,16,17,18,19,20,21].

Takahashi *et al.* (1979) [22] extracted water-soluble and insoluble homoglucans from *R. crassa* Delise ex Nyl. (currently *R. siliquosa* (Huds.) A.L. Sm.). Separately, from the mycobiont, the hydrolysis of homoglucan produced glucose (**3**) similar to the symbiotic association, although galactose (**4**) was produced from the alga (phycobiont) itself by hydrolysis of its galactomanan [22]. Polysaccharides of *R. sinensis* were hydrolyzed with sulfuric acid and monosaccharides were derivatized with 1-phenyl-3-methyl-5-pyrazolone (PMP) producing the monosaccharides glucose, mannose (**5**), rhamnose (**6**) and galactose in a molar ratio of 5.05:3.89:0.14:0.09 [23].

Kosugi *et al.* [24] isolated d-arabitol (**7**) from the green alga *Trebouxia* sp., photobiont from *R. yasudae* lichen. The aqueous extract of the lichen stem produced several sugars, among them d-arabitol, which was identified by nuclear magnetic resonance (NMR), mass spectrometry (MS) and gas chromatography (GC) [24]. From the extract of the *R. fraxinea* stem in acetone, d-arabitol and mannitol (**8**) were isolated, and from the aqueous extract glucose, galactose, glucosamine (**9**), arabinose (**10**), xylose (**11**), rhamnose and glucuronic acid (**12**) were obtained [25]. d-Arabitol was also isolated from the species *R. reticulata* [26], *R. calicaris* and *R. sinensis* [27], *R. siliquosa* [28], *R. tayloriana* [29], *R. geniculata* and *R. scopulorum* [30].

Six species of *Ramalina,* and separately, phycobionts and mycobionts of some of them, were studied [14,16,17,18,19,20,21,31,32], where it was concluded that all the studied species presented components structurally similar to the ones from the isolichenan, laminaran, nigeran and galactomannan classes [18]. These include the species, *R. usnea* (L.) R. Howe (currently, *R. australiensis* Nyl.) [21,32,33], *R. ecklonii* (Spreng.) Meyen & Flot. (currently *R. celastri*) [14,16,17,33,34], *R. dendriscoides* Nyl. [18], *R. fraxinea* (L.) Ach. [18], *R. gracilis* (Pers.) Quél. [18,20,31] and *R. peruviana* Ach. [18,19]. From the *R. complanata* mycobiont were extracted the mentioned polysaccharides, in addition to a type of β-glucan, a lentinan (**13**) and a heteropolysaccharide whose composition is Man:Gal:Glc in the ratio of 21:28:51 [35].

From the alga *Trebouxia puymaly*, a photobiont of *R. gracilis,* a polysaccharide containing bonds (1→5)-β-galactofuranosyl as the main chain was extracted, with replacement at *O-*6 by β-gal*f* units. Amylose (**14**) has also been found in small quantities in this photobiont and also in the symbiont of *R. celastri* (Spreng.) Krog & Swinscow, probably originating from its photobiont [16,17,20]. These polysaccharides were not found in symbiotic stems of the species *R. gracilis* [20]. However, several complex side chain structures, composed primarily of D-Man*p* units were found with replacements at the positions *O-*4, *O*-2,4, *O*-2,3 and *O*-3,6 [20,31]. In the *Trebouxia* phycobiont stems of the species *R. maciformis* starch was found distributed in the chromatophores [36].

The lichen species of Antarctic *R. terebrata*, whose photobiont is a *Trebouxia* species, produced hemicelluloses and cellulose/lignin. Neutral and acidic monosaccharides components were derivatized with tetramethylsilane (TMS), producing glucose as most abundant neutral monosaccharide, but also the derivatives revealed the presence of galacturonic acid (**15**) as the most abundant among fatty acids [37].

Komiya and Shibata (1971) [38] studied the metabolism of polyols of *R. crassa* and *R. subbreviuscula* from phyco and mycobionts grown, and observed that ribitol (**16**) was produced by phycobiont and was converted to arabitol and mannitol in the mycobiont [38]. The principal *Ramalina* polysaccharides are compiled in Figure 1 and Table S1 (supplementary material).

### 2.2. Usnic Acid and Derivatives

One of the most known, isolated and discussed compounds of lichenized fungi is usnic acid (**17**). It was isolated for the first time in the year 1834, by Rochleder *et al.*, from among others, the species *R. calicaris* (L.) Röhl. [3]. However, all species of the genus *Ramalina* contain usnic acid in variable concentration [5,39]. Studies of the isolation and biological activity tests of the compound have presented, almost always, unexpected results, generating numerous publications [5,7,10,12,13,16,21,25,26,27,28,29,34,40,41,42,43,44,45,46,47,48,49,50,51,52,53,54,55,56,57,58,59,60,61,62,63,64,65,66,67,68,69,70,71,72,73,74,75,76,77,78,79,80,81,82,83,84,85,86].

Lee *et al.* [8] isolated from *R. terebrata* Hook. f. & Taylor, a species from the Antarctic, usnic acid derivatives known as usimine A (**18**), B (**19**) and C (**20**), [8,10,11], and the last one presented good anti-proliferation activity results on human dermal fibroblasts [8,11]. González *et al.* (1991) [12] isolated (+)-*iso*-usnic acid (**21**) from the lichen *R. hierrensis* [12], and Asahina and Fukuziro (1932) [13] isolated usninic acid (**22**) from *R. calicaris*, shown in Figure 2 and Table S2 (Supplementary Material).

### 2.3. Depsides

Among the isolated depsides, sekikaic acid (**23**) was found in 26 species of *Ramalina* [1,25,27,40,43,46,50,51,54,55,57,59,60,61,62,63,65,87,88,89,90,91,92,93,94,95,96], atranorin (**24**) in 19 species [1,12,28,55,57,59,60,64,66,67,72,83,87,92,95,97], divaricatic acid (**25**) was found in 11 species [12,43,49,57,59,60,98] like homosekikaic acid (**26**) [60,87,89,90,92,96]. Ramalinolic acid (**27**) was found in ten species [1,43,46,60,80,87,88,99], obtusatic acid (**28**) appears in seven species [1,13,25,44,50,68,77,80,88,100] and the depside chlorinated tumidulin (**29**) was found in six species [64,73,101,102], like 4′-*O*-demethylsekikaic (**30**) [1,60,87] and evernic acids (**31**) [1,13,44,50,54,65,68,81,88,100,103]. 4′-*O*-Methylnorhomosekikaic acid (**32**) [60,89,104] was found in five species.

Some compounds were found in a smaller number of species, usually three, two or one. 4′-*O*-methylnorsekikaic (**33**) [1,40,60], 2′-*O*-methylsekikaic (**34**) [1,60,104,105] acids and chloroatranorin (**35**) [2,55,66] were found in three species. Cryptochlorophaeic (**36**) [55,60], 4′-*O*-demethylhomosekikaic (**37**) [60], diffractaic (**38**) [46,66], 4-*O*-demethylbarbatic (**39**) [1,106,107], and ramalinaic acids (**40**) [1,108] were found in two species.

In just one species, were found the tridepside gyrophoric (**41**) [46] and trivaric acids (**42**) in *R. americana* Hale [46,109], perlatolic acid (**43**) in *R. stenospora* Müll. Arg. [55], 4-*O*-demethylnorhomosekikaic acid (**44**) [60] in *R. peruviana* [56], 4′-*O*-methylsekikaic (**45**) [60], 4′-*O*-methylpaludosic (**46**) [60,104], 4,4′-di-*O*-methylcryptochlorophaeic (**47**) [104] and boninic (**48**) [76] acids were found in *R. asahinae*, stenosporic acid (**49**) in *R. stenospora* [1,55], 5-hydroxysekikaic acid (**50**), new hydroquinone depside, in *R. farinacea* [62] and 5-chlorosekikaic acid (**51**) in *R. glaucescens* [78]. From *R. leiodea* [110] was isolated olivetoric acid (**52**), and paludosic acid (**53**) in *R. paludosa* [72]. An orcinol-type meta-depside with an oxidized side chain 4-*O*-methyloxo-cryptochlorophaeic acid (**54**) [111] was isolated from *R. subfraxinea* [111], lecanoric acid (**55**) from *R. lacera* [46] and aliphatic depside bourgeanic acid (**56**) found among other species in *R. bourgeana* [102,112,113]. See Figure 3 below and Table S3 in the Supplementary Material.

### 2.4. Depsidones

The most common compound of the depsidones class among species of *Ramalina* was salazinic acid (**57**), identified in 27 species [1,28,39,40,41,46,51,54,55,56,59,60,61,70,79,80,88,89,98,106,114,115], followed by norstictic acid (**58**) in 15 species [1,5,39,42,46,50,60,61,70,88,91,115,116]. Hypoprotocetraric acid (**59**) was found in six species [1,5,28,39,115,117], scopuloric acid (or stictic) (**60**), in five species [5,12,28,39,41,57,80,88], and the protocetraric acid (**61**), in four species [1,5,28,39,42,46,50,70,88,106,115].

Some depsidones were found in just one species, such as connorstictic acid (**62**) in *R. anceps* Nyl. [60], cryptostictic acid (**63**) found in *R. cuspidata* in the varieties *cuspidata* and *armorica* [5], peristictic acid (**64**) in *R. cuspidata* var. *armorica* [5], conhypoprotocetraric acid (**65**) in *R. siliquosa* var. x [5], variolaric acid (**66**) and gangaleoidin (**67**) in *R. hierrensis* [12], physodic acid (**68**) in *R. leoidea* [110] and the coquimboic acid (**69**) found in *R. tumidula* [118]. See Figure 4 below and Table S4 in the Supplementary Material.

### 2.5. Fatty Acids

Lichenized fungi contain many of the fatty acids commonly found in higher plants [119] and in marine natural products [120]. Among these fatty acids, oleic (**70**), palmitic (**71**) and stearic (**72**) acids were found in *R. lacera* [46], *R. yasudae* [119] and in the mycobiont of *R. celastri* [121]. α-Linolenic acid (**73**) was found in *R. yasudae* [119] and *R. lacera* [46]; and linoleic (**74**) and myristic (**75**) [119] acids were found in *R. yasudae* [45]. Arachidonic acid (**76**) [119] was not found in the symbiont of *R. yasudae*, but it was present in small quantity in the photobiont *Trebouxia* and traces of its mycobiont [119]. The long chain γ-lactone acids D-protolichesterinic (**77**) and nephrosterinic (**78**) were obtained from *R. almquistii* Vain. [44,122] and protolichesterinic acid was also found in *R. roesleri* [92]. See Figure 5 below and Table S5 in the Supplementary Material.

### 2.6. Other Compounds

Among other classes of compounds found in lichens of the genus *Ramalina*, the research of Czeczuga and Ferraro (1987) [123] presents isolated carotenoids of lichens from the Argentinian species *R. ecklonii* (a) and *R. usnea* (b) and the review of Dembistsky (1992) [124] features carotenoids of lichens from New Zealand, among them the species *R. celastri* (c), the isolated compounds were β-cryptoxanthin (a,b) (**79**), lutein epoxide (a,b,c) (**80**), violaxanthin (a,c) (**81**), auroxanthin (a,b) (**82**), astaxanthin (b,c) (**83**), mutatoxanthin (b) (**84**), lycoxanthin (a) (**85**), antheroxanthin (a,b) (**86**), ε-carotene (b) (**87**), zeaxanthin (a,c) (**88**), β-carotene (c) (**89**), α-doradexanthin (c) (**90**), lutein (b,c) (**91**), hydroxyechinenone (a) (**92**), diatoxanthin (a) (**93**), neoxanthin (a,b) (**94**) and rhodoxanthin (a,b) (**95**) [123,124]. Several of these carotenoids are also found in higher plants, in algae, yeast and other marine organisms [120]. See Figure 6 below and Table S6 (Supplementary Material).

Some steroids isolated from lichens are also found in several marine organisms, including sponges, algae, among others, possessing important biological activities as antitumor agents and against *Mycobacterium tuberculosis*, such as ergosterol peroxide. β-Sitosterol, a substance widely found in higher plants, has antibacterial and antifungal properties and is an antihypercholesterolaemic, estrogenic and hypolipididemic agent [120].

β-Sitosterol (**96**) was found in *R. africana* [44] and *R. hierrensis* [12], brassicasterol (**97**) in *R. africana* [44] and *R. tingitana* [69], and lichesterol (**98**) [44] in *R. africana* [44]. Ergosterol peroxide (**99**) was isolated from *R. hierrensis* [12] and *R. tingitana* [69] and cerevisterol (**100**) was isolated from *R. hierrensis* [12]. The triterpenes ursolic acid (**101**) and *iso*-arborinol acetate (**102**) were found in *R. hierrensis* [12] and friedelin (**103**) in *R. ecklonii* [83], and the diterpenes (−)-sandaracopimaric acid (**104**) in *R. hierrensis* [12] and ceruchinol (**105**) in *R. tigrina* [125] and *R. ceruchis* var. *tumidula* [126]. Figure 7 is shown below and Table S7 in the Supplementary Material.

From *R. lacera* [46] were isolated the polar lipids diacylglyceryl-*N,N,N*-trimethylhomoserine (**106**) (DGTS), diacylglyceryltrimetylalanine (DGTA) (**107**), phosphatidylcholine (PC) (**108**), phosphatidyl-ethanolamine (PE) (**109**), phosphatidylinositol (PI) (**110**), phosphatidic acid (PA) (**111**) and the glycolipid sulfoquinovosyl diacylglycerol (SQDG) (**112**). The glycolipids monogalactosyldiacylglycerol (MGDG) (**113**) and digalactosyldiacylglycerol (DGDG) (**114**) were isolated both from *R. lacera* [46] and *R. celastri* [127]. From *R. celastri* was obtained a glycosphingolipid, *O*-β-d-galactopyranosyl-(1→1′)-ceramide (**115**) [128], which primary lipids components are (4*E*)-sphingenine, sphinganine and eicosasphinganine, esterified with palmitic, oleic and 2-hydroxypalmitic acids. From the stems of *R. fraxinea* were obtained a fraction containing the amines choline (**116**), betaine (**117**), histamine (**118**), acetylcholine (**119**) and β-phenethylamine (**120**) [25]. In *R. farinacea* the polyamines spermidine (**121**) and diamine putrescine (**122**) were detected [129,130] and in *R. calicaris* the polyamine spermine (**123**) [130]. Figure 8 below and Table S8 (Supplementary Material)

The amino acids found in lichenized fungi are similar to those found in many marine organisms and higher plants [120]. From *R. siliquosa* [131] and *R. fraxinea* [25], were isolated glutamic acid (**124**), aspartic acid (**125**), alanine (**126**), serine (**127**) and proline (**128**). Alanine was also found in *R. sinensis* [132]. The amino acids arginine (**129**), glycine (**130**), lysine (**131**), leucine (**132**), threonine (**133**), and glucosamine (**134**) were found in *R. siliquosa* [131] and the γ-aminobutyric acid (**135**) was found in *R. fraxinea* [25]. Others amino acids were also found in *R. siliquosa* [131] at very low concentrations. From *R. crassa* the amino acid taurine (**136**) was isolated [133]. See Figure 9 below and Table S9 in Supplementary Material.

From an Antarctic lichen species, *R. terebrata*, were isolated compound ramalin (**137**), a new hydrazide with antioxidant activity [9,10,11,134,135,136] and the cyclic depsipeptide stereocalpin A (**138**) [137,138,139].

Different phenolic compounds have been isolated from the species of the genus *Ramalina*. From *R. farinacea* was isolated 2,3-dihydroxy-4-methoxy-6-pentylphenylmethyl ester (**139**) [62], from *R. africana* were isolated divaric acid (**140**) and ethyl divaricatinate (**141**) [44], from *R. roesleri* 2-hydroxy-4-methoxy-6-propylbenzoic acid (**142**) and 2,4-dihydroxy-3,6-dimethylmethyl ester benzoic acid (**143**) [92] and from *R. dilacerata* was isolated isorhizonic acid (**144**) [140].

Organic acids and derivatives as well as fatty acids esters were found in *Ramalina* species. α-Crotonic acid (**145**) was isolated from *R. reticulata* [26] and abscisic acid (**146**) from *R. farinacea* [129], while from *R. fastigiata* [141] were isolated the esters ethyl caprilate (**147**), ethyl palmitate (**148**) and ethyl stearate (**149**). The compounds benzopyran divaricat acid (**150**) and anthraquinone (**151**) were found in *R. hierrensis* [12].

Aliphatic compounds and cycloaliphatic have been also found in lichens, especially alkanes with C_13_ and C_17_–C_40_ carbon chains [1]. The research of Zygaldo *et al.* [142] presented a composition of *n*-alkanes from 15 species of lichens belonging to six different families, including the Ramalinaceae family, among the species of this family *R. celastri* and *R. ecklonii* are found. The analyses results by GC/MS from the lichens stems showed the presence of *n*-alkanes with chain ranging from C_13_–C_40_ and showed that 14 *n*-alkanes with chain C_20_–C_33_ were common to all studied species. Branched alkanes have not been found. The composition of the *Ramalina n*-alkanes was characterized by a high level of C_29_ and C_31_ (>7%) in both species. Difference between the species were observed, because *R. ecklonii* presented a higher concentration of C_27_ (11.3%) and C_31_ (19.5%) against 5.6% of C_27_ and 13.2% of C_29_ for *R. celastri* [142]. The compound cycloaliphatic aspicilin (**152**) was isolated from *R. ecklonii* [73] and ethylene (**153**) extract in ethyl ether was found in *R. lacera* [1]. See Figure 10 below and Table S10 in the Supplementary Material.

## 3. Biological Activity

Lichens have been used as a promising biological source of metabolites with different bioactivities [1,2,53]. Huneck and Yoshimura [1] mention in their classic work the monograph of Zopf in 1907 about the pharmacological activities of lichens compounds. The authors also highlighted the following biological activities that have already been researched up to the present time: antibiotic activities, antitumor and antimutagenic activities, activities against human immunodeficiency virus (HIV), allergenic activities, growth inhibitory activities in plants, and enzyme inhibitory activities, among other activities [1]. However, only a very limited number of lichen compounds were tested for their biological activities and their therapeutic potential in medicine. This happens because of the difficulties in identifying the species, in the small amounts collected for such studies and difficult isolation of pure compounds for structural elucidation and biological activity testing [2].

The cause of the antibiotic activity of many lichens has been assigned to usnic acid [52], although there are authors that mention that the antibiotic activity of lichens is related to the presence of phenolic derivatives [3]. The mechanisms of antibiotic action of lichenic acids, more specifically usnic acid and its derivatives, suggest that these compounds modify the structures of proteins causing irreversible changes, and may even produce apoptosis [3]. This compound presents activity against bacteria, fungi and yeasts [1,2,3,42,52,53,88,114,143]. Beside the antimicrobial activity against human and plant pathogens, usnic acid (**17**) presents antiprotozoal action [53], analgesic and antipyretic [1,53], anti-inflammatory [53], antitumor and antimutagenic [2,3,53], antiviral [1,2,3,53] plant growth inhibiting [1] , enzyme activity inhibitory [1,2] and allergenic properties [1].

Another class of compounds, the polysaccharides, which can be from multiple sources (plants, fungi and lichens) have different biological activities, acting as antitumor and anti-inflamatory agents and immunomodulators [31]. Several studies have shown that many polysaccharides can act as biological response modifiers (BRM). This can happen by activation of the immune response involving macrophages, T helpers and natural killer cells (NK cells), T cell differentiation, proliferative response of polymorphnuclear cells, production of interleukins and interferon, as well as increasing the phagocytotic activity [34]. Some biological activities of extracts and compounds isolated from the genus *Ramalina* will be presented next.

### 3.1. Antimicrobial Activity

The development of new antibacterial compounds is an urgent issue to suppress the evolution of pathogenic bacteria rssistant to the available drugs [123]. Therefore, many studies have been developed in an attempt to discover new substances with this activity.

The usnic acid isolated from *R. reticulata* Kremp. (currently *R. menziesii* Taylor) presented activity against Gram (+) organisms and some acid resistant bacteria, including *Mycobacterium tuberculosis* (Zopf 1883) Lehmann and Neumann 1896, but not against a range of different Gram (−) organisms [114] in agreement with other performed studies [93].

Tay *et al.* [42] tested the antimicrobial activity of the acetone extract obtained from *R. farinacea* (L.) Ach. and its constituents usnic acid (**17**), norstictic acid (**58**) and protocetraric acid (**61**) against thirteen bacteria, two yeast and ten filamentous fungi. The extract demonstrated activity against six bacteria with concentrations ranging between 3.3–6.6 µg/25 µL and 3.3 µg/25 µL for the two yeasts tested *Candida albicans* and *Candida glabrata* and no activity against filamentous fungi. Regarding the compounds tested, the usnic acid showed the best results, and for the six bacteria on which it had an effect, the minimum inhibitory concentration (MIC) varied between 0.39–3.1 µg/25 μL; the less active was the norstictic acid, with a MIC ranging between 11.7–188 µg/75 µL. Protocetraric acid did not present action against fungi and bacteria. With the yeasts, usnic acid also presented the best results, with a MIC value of 0.05 µg/62.5 µL, while for norstictic acid the MIC value was 2.9 µg/75 µL and for protocetraric acid the value was 3.9 µg/75 µL. The study demonstrated that among the three compounds tested only usnic acid showed any significant activity at low concentration against the Gram (+) and fungi tested.

The antioxidant and antimicrobial properties of methanolic extracts from five lichenized fungi species were tested by Gulluce *et al.* [144], and the results showed that the extracts of *R. polymorpha* and *R. pollinaria* inhibited 10 and 11 bacterial species, respectively, from a total of 35 tested species. Assays were performed using the disk diffusion method and micro-dilution assay to obtain the values of MIC values (µg/mL). The *R. pollinaria* extract presented MICs between 5.62–62.5 µg∙µL^−1^. For both fungi tested, the MIC values of *R. polinaria* were 31.25–62.5 µg∙µL^−1^ for *Trichophyton rubrum* and *Sclerotonia minor,* respectively, while the MIC value of *R. polymorpha* was 62.5 µg∙µL^−1^ for both fungi. Data from this study indicated that there must be antimicrobial compounds in the tested extracts, which include the *Ramalina* genus [144].

Cansaran *et al.* [52] investigated the biological activities of five *Ramalina* species obtained in Turkey: *R. fastigiata* (Pers.) Ach., *R. capitata* (Ach.) Nyl., *R. polymorpha* (Lilj.) Ach., *R. pollinaria* (Westr.) Ach. and *R. fraxinea*. They used the agar disk diffusion method with a tetracycline as control. The study presented that the extracts of lichens showed antimicrobial activity at different rates and that the greater the concentration of usnic acid in the extract, the greater the inhibition of microorganisms. However, all the extracts inhibited *Bacillus subtilis*, with *R. fastigiata*, which presented a higher concentration of usnic acid among the extracts (about 3.3% by dry weight) showing greater inhibition. Only the *R. fraxinea* extract, with a usnic acid concentration of 0.17% did not inhibit *Bacillus megaterium*. *Enterococcus faecalis* and *Proteus mirabilis* were only inhibited by the extracts of *R. fastigiata*, with 3.3% of usnic acid and *R. capitata*, with 1.25% of usnic acid. Both of this extracts also inhibited *Escherichia coli* and beside them, *Ramalina polymorpha* extract, with 0.27% of usnic acid inhibited this bacteria with a lower inhibition rate. The extracts were especially active against Gram (+) bacteria, although none of them inhibited *S. aureus*. Among tested Gram (−) bacteria, neither of the extracts inhibited *Pseudomonas aeruginosa* and *Escherichia coli*, the bacteria *Proteus mirabilis* and *Escherichia coli* were inhibited by three and two of the extracts, respectively, with higher concentrations of usnic acid [52].

Crude extracts of the species *Ramalina hossei* Vain. produced in methanol, chloroform and petroleum ether solvents, were tested for their antimicrobial activity by the Kirby Bauer method. The results demonstrated that the extracts showed better activity against Gram (+) than against Gram (−) bacteria. Chemical tests of the extracts revealed the presence of usnic acid (**17**) and sekikaic acid (**23**) as a mixture. The extracts were more active against Gram (+) species, confirming the results of other studies, and the methanol extract showed greater inhibition of bacteria than other extracts [93].

In the study conducted by Babita *et al.* in 2008 [145], the antibacterial potential of methanolic extracts from five Antarctic lichens species belonging to four different genres were tested, among them the species *R. terebrata,* and it was shown that considerable antimicrobial activity were obtained against *Bacillus subtilis* (MIC 33.8 ± 0.15 µg∙mL^−1^ and IC_50_ 16.9 ± 0.1 µg∙mL^−1^) and *S.* aureus (MIC 85.7 ± 6.7 µg∙mL^−1^ and IC_50_ 42.9 ± 3.4 µg∙mL^−1^), but no activity were observed against *Candida albicans*, *Pseudomonas aeruginosa* and *Escherichia coli*; in this case the authors used the methodology of the sterile paper disk described by Bhattarai *et al.* in 2006 [146]. The MIC was determined by the broth dilution method described by Swenson *et al.* in 1982 [147]. The results showed strong antibacterial activity of the extracts against Gram (+) bacteria, indicating that these species of Antarctic lichens produce compounds with significant antibiotic properties [145].

The research of Paudel *et al.*, conducted in 2010 [134] reported that five compounds isolated from the methanolic extract of the Antarctic lichen *R. terebrata*, namely usnic acid (**17**) and the derivatives, usimine A (**18**), B (**19**), C (**20**) and ramalin (**137**), were tested against the bacteria *Staphylococcus aureus* and *Bacillus subtilis* by the disc diffusion method. All tested samples presented activity against *B. subtilis*, where the values of MIC of the isolated compounds ranged from 1–26 µg∙mL^−1^ for these bacteria. Only the crude methanolic extract and usnic acid showed activity against *S. aureus* [134].

In 2012, Paudel *et al.* [148] studied the antibacterial activity of twenty-four lichens species of six lichen families from Nepal, among them *Ramalina* spp. Twenty one species were active against *B. subtilis* and seven were active against *S. aureus.* The results showed that *Ramalina* spp. presented MIC values of 8.5 and 15.1 µg∙mL^−1^ for *B. subtilis* and 65.3 µg∙mL^−1^ for *S. aureus*, while the MICs of the commercial product ampicilin, used as control, were 0.4 and 0.35 µg∙mL^−1^ for the respective tested bacteria. These results confirm those of other studies involving lichen species of the same genus [142]. The data showed a strong potential of these extracts as antibacterial agents. The results obtained by Sisodia *et al.* with hexane extract of *R. roesleri* confirmed the high activity against *S. aureus*, and also against *Streptococcus mutans* [75].

### 3.2. Antioxidant Activity

Several species of lichens from different genres and regions of the World have antioxidant potential [96]. Gulluce *et al.*, in 2006 [144], showed that the methanol extracts of *R. pollinaria* and *R. polymorpha* species did not show antioxidant properties by the diphenylpicrylhydrazyl method (DPPH), however, a low inhibition was exerted on the oxidation of linoleic acid in the linoleic acid/β-carotene oxidation method obtaining a percentage inhibition (I%) of 26 ± 1, 19 ± 2 and 96 ± 1 for *R. pollinaria*, *R. polymorpha*, and for the control compound butylated hydroxytoluene (BHT), respectively, so it was concluded that the extracts of these species showed little antioxidant potential [144].

Kumar *et al.* [94] studied the methanol extracts of two lichens of the Ramalinaceae family, *R. conduplicans* and *R. hossei*, and evaluated their antioxidant activity by the DPPH method and by the reduction of Fe^3+^ assay. At the concentration of 250 µg/mL an elimination of 56.11% and 48.04% occurred for *R. hossei* and *R. conduplicans,* respectively, and at 500 µg/mL the elimination was 61.53% and 59.01% and with 1000 µg/mL it was 79.05% and 72.63%, below the control values of ascorbic acid, which were 92.52%, 95.12% and 97.33% at the same concentrations. *R. hossei* showed a higher free radical elimination rate than *R. conduplicans.* The substances usnic acid (**17**), sekikaic acid (**23**), salazinic (**57**) acid and tannins were detected in the lichen methanol extracts, which showed promising antioxidant potential results [94].

The research of Luo *et al.* [96] presented data on the antioxidant activity of the lichen *Ramalina conduplicans*. The free radical elimination activity of the lichen methanol extract was tested by the Blois method (1958, [149]), using DPPH and the result was presented as IC_50_. An assay of the linoleic acid peroxidation activity using the thiocyanate method proposed by Mitsuda *et al.*, in 1996 [150], with some modifications proposed by Luo *et al.* in 2009 [151] was also performed. The percentage of inhibition was found to be 55.8% at the concentration of 330 µg∙mL^−1^, presenting an IC_50_ of 0.232 mg∙mL^−1^, relatively low compared to data from other lichen species tested. The extract exhibited high antioxidant activity against linoleic acid peroxidation, 85.2% at a concentration of 2.0 mg∙mL^−1^, which was higher than the inhibition of ascorbic acid used as control at the same concentration. The main compounds detected by bioauthographic thin layer cromatography (TLC), high performance liquid chromatography (HPLC) and ultraviolet (UV) spectroscopy were sekikaic acid (**23**) and homosekikaic acid (**26**) depsides. The IC_50_ values of the pure compounds were 0.082 mg∙mL^−1^ for sekikaic acid and 0.276 mg∙mL^−1^ for homosekikaic acid, demonstrating that these compounds are promising antioxidants [96].

Yim [10] has patented a pharmaceutical composition containing ramalin (**137**) as an active ingredient for functional foods used as anti-aging products, cosmetics for skin whitening and as an anti-wrinkle agent since ramalin present better antioxidant effects than conventional commercially available antioxidants [10].

Ramalin (**137**) [9,10,134,135,136] was isolated from the water-methanol extract of *R. terebrata* by several chromatographic methods by Paudel *et al.* in 2011 [9]. The experimental data showed that this substance was five times more powerful (IC_50_ 0.99 ± 0.08 µg∙mL^−1^) that the commercial drug BHT (IC_50_ 4.98 ± 0.9 µg∙mL^−1^) in the elimination of free radicals by DPPH method, and twenty-seven times more powerful in eliminating free radicals by the 2,2′-azino-bis(3-ethylbenzothiazoline-6)-sulfonic acid (ABTS^+^) method than the analogous compounds vitamin E and Trolox, and two and a half times more potent than BHT when used for reducing Fe^3+^ ions to Fe^2+^. Ramalin also proved to be 1.2 times more powerful than ascorbic acid in the elimination of superoxide radicals. The *in vitro* tests of the antioxidant activity showed that 1.0 µg∙mL^−1^ significantly reduced nitric oxide (NO) produced and 0.125 µg∙mL^−1^ reduced the production of hydrogen peroxide (H_2_O_2_) in lipopolysaccharides (LPS) stimulated in murine macrophage cells Raw 264.7. Considering the data set, it is verified that ramalin is a therapeutic candidate for the control of the oxidative stress in cells, since the compound has very low or no cytotoxicity on human keratinocytes and fibroblasts cells at its active antioxidant concentrations [9].

Halici *et al.* [152] studied the antioxidant and gastroprotective activities of ethanol-water extracts (1:1), ethanolic, methanolic and aqueous extracts from the lichen *Ramalina capitata*. The results showed that the extracts significantly reduced gastric injuries induced by indomethacin. The most significant gastric protective effect was obtained with ethanol-water extract (1:1) (66.6%) at a dose of 200 mg∙kg^−1^. Indomethacin caused significant decreases in the levels of glutathione peroxidase (GP_x_), glutatione S-transferase (GTS), superoxide dismutase (SOD) and reduced glutathione (GSH). However, ethanol–water extract (1:1) showed significant antioxidant activity against oxidative damage in the stomach tissues, increasing the levels of GP_x_, GST, SOD and GSH. The catalase and myeloperoxidase levels increased by the indomethacin were lower in the groups administered with ethanol-water (1:1) extract. Furthermore, it can be observed that all tested extracts presented significant antioxidant activity *in vitro*, with 64.9% for the ethanol-water extract, 52.2% for the ethanolic extract, 56.7% for the methanolic extract and 73.7% for the aqueous extract in the assay of linoleic acid peroxidation inhibition. However, the antioxidant activity of these extracts was lower than that of the compound Trolox, used as control, but was higher than that of ascorbic acid. These results indicate that *R. capitata* extract had gastroprotective effects against gastric ulcer [152].

Four lichen genres (*Ramalina*, *Parmotrema*, *Bulbothrix* and *Cladia*) collected in Malaysia were studied by Stanly *et al.* [153] and had their antioxidant activities compared by the DPPH method. The *R. peruviana* species presented higher activity in the elimination of free radicals (86%) with its extract in acetone at a concentration of 750 µg∙mL^−1^. *R. peruviana* also gave the lowest effective concentration needed to eliminate 50% (EC_50_) of free radicals with the extract in acetone presenting and EC_50_ of 60.66 µg∙mL^−1^ among all four lichens species tested. For the assay using the β-carotene bleaching method, the best activity was obtained by the extract in acetone of *Bulbothix isidiza* (66.7%), followed by the acetone extract of *R. peruviana* (57.3%), yet the level of phenolic compounds found for the *R. peruviana* extract was the lowest among the species tested, demonstrating that there is no correlation between the total phenolic content and antioxidant activity [153].

The study developed in 2012 by Paudel *et al.* [148] presented data on the antioxidant activity of methanol-water extracts (8:2) from 24 Nepal lichen species. All species tested and the commercial product butylated hydroxyanisole (BHA) used as control showed free radical scavenging capacity by the DPPH, ABTS^+^ and Fe^3+^ reduction methods at a dose dependent concentration. For the DPPH method, the inhibitory concentration 50% (IC_50_) ranged from 5.6–98.6 µg∙mL^−1^ for the extracts, wherein the NL-17 and NL-18 samples from *Ramalina sp*. presented IC_50_ of 32.9 and 8.7 µg∙mL^−1^, respectively, while the IC_50_ of the control BHA was 3.5 µg∙mL^−1^. The results of tests with ABTS^+^ confirmed the DPPH data, with the IC_50_ values ranging from 6.9–99.8 µg∙mL^−1^, and for the samples NL-17 and NL-18 the IC_50_s were 52.0 and 33.2 µg∙mL^−1^ and for the compound Trolox a vitamin E analog used as a control it was 46.4 µg∙mL^−1^, so the compounds showed strong ability to eliminate free radicals, having great oxidant activity.

The antioxidant activity of extracts and isolated compounds from the lichen *R. roesleri* were assessed by Sisodia *et al.* [92] by the DPPH method. The results showed a range of free radical elimination power in the extracts between 29.42%–87.9%. The compounds atranorin (**24**), protolichesterinic acid (**77**), usnic acid (**17**), 2-hydroxy-4-methoxy-6-propylbenzoic acid (**142**), homosekikaic acid (**26**), sekikaic acid (**23**), 2,4-dihydroxy-6-propylbenzoic acid (**142**) and 2,4-dihydroxy-3,6-dimethylbenzoate (**143**) were isolated from the hexane extract. Among the compounds, the best antioxidant activity was exhibited by sekikaic acid, followed by homosekikaic acid [92].

### 3.3. Antiviral Activity

Few studies were found on the antiviral effects using extracts or pure compounds isolated from lichens. The research of Fazio *et al.* [48] evaluated the antiviral and cytotoxic activity effects against Vero cells infected with arenavirus Junin (JUNV), causative agent of hemorrhagic fever on human beings in Argentina and against arenavirus Tacaribe (TCRV), a non-pathogenic member of the Arenaviridae family, of two secondary metabolites obtained from mycobiont cultivation of two genera of lichens, *Tlechoschistes chrysophthalmus* and *Ramalina celastri*. The antiviral and virucidal activity of usnic acid, a metabolite isolated from *R. celastri*, the subject focus of this review, will be presented. Parientin, a compound isolated from *T. chrysophthalmus* will not be discussed. Antiviral activity testing was performed using concentrations lower than the 50% cytotoxic concentration (CC_50_), for usnic acid (65.1 µM). The results demonstrated that usnic acid (**17**) reduced the production of Junin virus in infected Vero cells in a dependent dose manner, and 50% inhibition was obtained at an effective concentration (EC_50_) of 9.9 µM. Regarding the TCRV arenavirus, the effective concentration was 20.6 µM. The selectivity indexes (CC_50_/EC_50_) of usnic acid for JUNV and TCRV arenavirus were 6.8 and 3.2, respectively, indicating a specific antiviral activity against these viruses and not just a general consequence of its action on cellular toxicity. In order to test the viability of virus inactivation by the direct effect on the viral particles, one virucidal assay was performed using the methodology proposed by Garcia *et al.* in 2002 [154]. When suspensions of JUNV or TCRV particles were incubated with usnic acid before cell infection, any remaining difference in infectivity of virus suspensions was detected between treated and untreated cells, so the virus-inhibitory effect observed in the inhibition assay performance was due to a real antiviral activity, exercised during the multiplication of the virus in the host cell [48].

Esimone *et al.* [155] obtained from *R. farinacea* lichen species a fraction soluble in ethyl acetate (ET4) which inhibited infection by adenoviral and lentiviral vectors such as HIV-1 type. Herpes virus type 1 (HSV-1) and respiratory syncytial virus (RSV) inhibition were also evaluated with this fraction. The anti-HIV and anti-HSV activities were quantified by the response of the β-galactosidase expression from the lineages of the indicator cells, while the anti-RSV activity was determined by an immunofluorescence technique. The ET4 effect on enzymatic activity of HIV-1 reverse transcriptase was also evaluated by chemiluminescence. It was demonstrated that the fraction strongly inhibited HSV-1 and RSV, with IC_50_ values of 6.09 and 3.65 µg∙mL^−1^, respectively. The fraction inhibited reverse transcriptase with an IC_50_ of 0.022 µg∙mL^−1^. Bioassay guided ET4 fractionation lead to a subfraction rf0 that showed activity against lentiviral vector and HIV-1 (RNA virus), but not against HSV-1 (DNA virus) and the rfM subfraction showed activity against HSV-1 but not against the lentivirus vector. Therefore, the study showed that the *R. farinacea* ET4 fraction has antiviral activity against DNA viruses (adenovirus, HSV-1) and against RNA viruses (HIV-1 and RSV) [155].

Recently, Lai *et al.* [62] studied the effect of *R. farinacea* lichen phenolic compounds from Nigeria on respiratory syncytial virus. In a preliminary test, the results showed that the lichen extract inhibited virus development. From the thirteen phenolic compounds isolated and evaluated against the syncytial virus, sekikaic acid (**23**) presented a higher inhibition to the virus RG lineage, with a 50% inhibitory concentration (IC_50_) of 5.69 µg∙mL^−1^, and for the strain A2 lineage the IC_50_ was 7.73 µg∙mL^−1^. The effect of sekikaic acid on HEp2 cells viability and Vero cell lineages was also investigated and the time addition assay showed that sekikaic acid interferes with viral replication in the virus post-entry stage 4 hours after virus addition, and the compound was 1.3 times more active than ribavirin used as negative control. The study concluded that although other compounds also showed antiviral inhibitory activity, sekikaic acid proved to be a powerful antiviral agent, with a selectivity index (SI) of 5.16 [62].

### 3.4. Antitumor and Cytotoxic Activity

The action of lichen-derived compounds on tumor cells has been a focus of reviews for a few decades [156]. Chemoprevention is a pharmacological approach used to prevent or reverse the carcinogenesis process. Natural products are among the agents used in chemoprevention, since many of these primary and secondary metabolite phytochemicals do not present toxicity to normal tissues and are known to have anticancer effects [157].

Hirayama *et al.* [122] tested the antitumor activity of 44 fractions adsorbed on cationic and anionic resins, extracted with hot water, nine lichens and 20 metabolites and their degradation products against ascites, an Ehrlich carcinoma solid type. The results showed that an adsorbed fraction from *R. almquistii*, and the compounds d-protolichesterinic acid (**77**) and nephrosterinic acid (**78**) were effective against Ehrlich carcinoma [122].

The cytotoxic activity of aqueous, ethanolic, chloroformic and *n*-hexane extracts from the lichen *R. farinacea* was evaluated by Esimone and Adikwu [45] using *Artemia salina*, a specie of saltwater crustacean. The crustacean eggs were incubated in seawater (collected in the Atlantic Ocean beach bar, Lagos, Nigeria), and allowed to incubate for 48 h at 28 °C. After incubation, 10 larvae (nauplii) of *Artemia salina* were introduced into vials containing growing concentrations of lichen extracts in the 10–1000 µg∙mL^−1^ range. After 24 hours, the number of surviving shrimp in each concentration of the extract was counted and the data were analyzed using the Finney program for determining the lethal concentration 50% (CL_50_) with a 95% confidence interval. The ethanolic extract showed higher cytotoxicity, with a CL_50_ of 6.0 µg∙mL^−1^ (IC 0.8–9.3), followed by the hexane extract with CL_50_ 11.3 µg∙mL^−1^ (IC 6.4–15.2), dichloromethane with a CL_50_ of 16.8 µg∙mL^−1^ (IC 11.9–26.3) and low cytotoxicity was observed in the aqueous extract, with a CL_50_ of 206.9 µg∙mL^−1^ (IC 91.5–389.2). The results show that the lichen extracts are promising sources of bioactive substances [45].

Stuelp-Campelo *et al.* [34] observed an effect of α-d-glucan from *R. celastri* on peritoneal exudate cells using a sarcoma-180 cells (S-180) *in vivo* assay. They found that the tumors developed in animals treated with glucan, at a dose of 200 mg∙kg^−1^, decreased by 80% in the control group [34]. The research of Leão *et al.* [158] presented a antitumor activity of α-d-glucan polysaccharides with (1→3)(1→4) bonds extracted from *R. celastri* and their sulfated derivatives that had as objective observing morphological alterations in HeLa cells using transmission electron microscopy (TEM). Although the α-d-glucan changed the cell volume, cytoplasmic density and mitosis, the resulting monolayer was similar to the results in the control. Microscopic analysis of cytoplasmic vesicles showed the presence of an eletrodense-free amorphous material in the cytoplasm and inner membranes. However the injury caused by secondary sulfate polysaccharide was evident, causing changes in cell adhesion and causing cells aggregation. Nuclear modifications such as fragmentation and chromatin condensation under the envelope suggest the occurrence of apoptotic cell death [158].

Bézivin *et al.* [47] carried out work on the *in vitro* cytotoxic activity of 24 extracts from five lichens species in two murine cells lineages (L1210-lymphocytic leukemia and 3LL-Lewis lung carcinoma) and four human cell lineages (K-562-chronic myelogenous leukemia; U251-glioblastoma; DU145-prostate carcinoma and MCF7-breast adenocarcinoma). Some extracts, among them the *R. cuspidata* (Ach.) Nyl. one, show interesting activities, particularly in the cell lineages K-562, U251, DU145 and MCF7 and with a good selectivity index. The ((3-[3,4-dimethylthiazol-2-yl]-2,5-diphenyltetrazolium bromide) (MTT) assay indicated significant cytotoxicity to three cell lineages with the hexane, diethyl ether and methanolic extracts of the lichen *R. cuspidata*, with IC_50_ (µg∙mL^−1^) ± standard deviation (SD) and selectivity index (SI) values for the lineages L1210 of 5.8 ± 1.9 µg∙mL^−1^ and 8.9; for 3LL 5.7 ± 0.6 µg∙mL^−1^ and 9.0; for DU145 6.7 ± 2.9 µg∙mL^−1^ and 7.7; for MCF-7 31.4 ± 11.2 µg∙mL^−1^ and 1.6; for K562 28.1 ±10.6 µg∙mL^−1^ and 1.8; and for U251 11.0 ± 7.5 µg∙mL^−1^ and 4.7 for the hexane extract, being the better results obtained from the three tested extracts. The results showed strong *R. cuspidate* cytotoxic potential, indicating that it must contain promising compounds against human cancer cells lines [47].

Haraldsdóttir *et al.* [159] tested the anti-proliferative effect against twelve human cancer cells lineages, of three lichen-derived substances—protolichesterinic (**77**), lobaric and baeomycesic acids—besides a commercial compound used as a specific inhibitor 5-lipoxygenase (LOX), zileuton. All tested compounds presented 5-lipoxygenase inhibitory activity and the compounds protolichesterinic and lobaric acid also inhibited 12-lipoxygenase. Compound **77** presented great inhibitory effects against all the cell lineages, with EC_50_ values ranging between 2.4–18.1 µg∙mL^−1^, showing the best result for the pancreatic cancer cell lineages Capan-1 and PANC-1 with EC_50_ 2.4 and 3.1 µg∙mL^−1^, respectively, and prostate adenocarcinoma PC-53, with EC_50_ 2.6 µg∙mL^−1^. In all tested lineages, protolichesterinic acid was more active than the control zileuton, showing the potential that this compound has against these human cancer cell lineages [159].

The study of Koparal *et al.* [160] evaluated the *in vitro* cytotoxic activity of (+)-usnic acid and (−)-usnic acid in human lymphocyte cells A549 from lung epithelial carcinoma and V-79 Chinese hamster lung fibroblast cells lineages. The tests were performed using the MTT methodology and the cytokinesis blocked micronucleus (CBMN) assay. The results showed that both enantiomers are not genotoxic, demonstrated by the lack of micronucleus induction and have significant cytotoxic and apoptotic effects in both cell lineages. Even at low doses, (+)-usnic acid showed high cytotoxic activity against cells. The results of MTT and the values of cell proliferation index (CPI), based on the results of the CBMN test obtained good agreement [160].

Einarsdóttir *et al.* [161] studied the mechanism of action of the usnic acid enantiomers against the breast cancer cell lineages T-47D and pancreatic cancer Capan-2 cells and also the proliferation, growth, and cell death effects. Both enantiomers exhibited similar anti-proliferative effects against both cell lineages. The IC_50_ values were 4.2 µg∙mL^−1^ and 4.0 µg∙mL^−1^ for (+)- and (−)-usnic acid against T-47D, and 5.3 µg∙mL^−1^ and 5.0 µg∙mL^−1^ against Capan-2, respectively. The other tests were performed only with the (+)-usnic acid. Proliferation assays showed that usnic acid at the concentration of 5.0 µg∙mL^−1^ caused a reduction in both cell lineages. The inhibitory effect on cell cycle was also confirmed by cytometric flow analysis, where the cells exposed to usnic acid showed a reduction in S-phase and few cells entering the G2/M phase, so the tests indicated that usnic acid affects the growth of the cells and their proliferation [161].

Bačkorová *et al.* [162] studied the cytotoxic/proliferative effect of four lichen secondary metabolites—parietin (**151**), atranorin (**24**), usnic acid (**17**) and gyrophoric acid (**41**)—against nine human cancer cell lineages. The best results of the MTT assay were obtained by usnic acid with HL-60 (promyelocytic leukemia), A2780 (ovarian carcinoma) and Jurkat (T cell lymphoblastic leukemia) lineages, presenting IC_50_ values of 48.5, 75.9 and 76.3 µg∙mL^−1^, respectively, being the most effective compound against all tested cells. Parientin showed cytotoxicity against HL-60 cells, Jurkat, HCT-116 p53^−/−^ (colon carcinoma submanifold without p53) and A2780, with IC_50_ values of 93.5, 181.6, 197.5 and 197.9 µg∙mL^−1^, respectively, and gyrophoric acid showed effect against HL-60 and A2780, with IC_50_ values of 146.7 and 198.3 µg∙mL^−1^. In a clonogenic assay usnic acid and atranorin in concentrations of 100 and 200 µM were the most effective compounds, significantly inhibiting the cloning capacity of all tested tumor cells. Regarding viability assays and quantifying the number of floating cells, usnic acid at a 50 µM concentration completely damaged A2780 cells and seriously affected HL-60 cells. Atranorin and gyrophoric acid also showed high cytotoxicity at tested concentrations of 100 and 200 µM. The research has also shown the preferential effect on the cell distribution and accumulation in S-phase of usnic acid, atranorin and gyophoric acid through a cell cycle assay with four cell lines. Usnic acid and atranorin were also the most effective compounds in the apoptosis induction in the four cell lines, followed by gyrophoric acid, and parientin was effective only in the HCT-116 p53^−/−^ lineage [162].

In the research carried out by Bačkorová *et al.* in 2012 [163] the cytotoxic mechanism results from the same metabolites parientin, atranorin, usnic acid and gyrophoric acid in the indiction of apoptosis in the human cancer cell lines A2780 and HT-29 were presented using five different methods from those used in 2011. The test results of the metabolites’ effect on the mitochondrial membrane potential (MMP) showed that usnic acid produced the greatest inhibition in both cell lines, at both concentrations of 50 and 100 µM. Atranorin produced high inhibition in A2780 at both concentrations (100 and 200 µM), but only produced a significant inhibition in the HT-29 lineage at a concentration of 200 µM. Gyrophoric acid gave good inhibition of A2780 at a concentration of 200 µM and parietin did not cause any cell lineage inhibition. In a phosphatidylserine externalization test, atranorin and usnic acid were effective at both concentrations tested, 50 and 100 µM, in HT-29 and A2780 cells. Gyrophoric acid was effective against A2780 at 200 µM and parietin caused no effect in both cells. In general, the A2780 cells were more sensitive than HT-29 cells. Regarding the reactive oxygen species (ROS) and nitrogen (RNS) assays, atranorin produced no increase of ROS in either cell line, but produced a significant increase in the production of RNS in both cells. Gyrophoric acid produced a ROS and RNS increase in both cells, but was more effective against HT-29. Caspase-3 activation had usnic acid as the most powerful inducer in HT-29 cell lineage, followed by atranorin and gyrophoric acid. Parietin did not cause significant activation changes of these proteins. Based on the detection of protein expression, it was shown that usnic acid and atranorin are programmed cell death activators in A2780 and HT-29 cells, probably by the mitochondrial pathway. In general, it can be concluded that usnic acid and atranorin are more effective at inhibiting cell proliferation and induce cells death more effectively compared to parietin and gyrophoric acid. The study demonstrated specific programmed cell death mechanisms induced by lichen secondary metabolites [163].

Brandão *et al.* [156] evaluated the cytotoxic activity of nine compounds isolated from seven different lichen species, including the *Ramalina* genus, among which seven of the nine compounds have been isolated in the genus *Ramalina*, namely atranorin (**24**) and usnic (**17**), diffractaic (**38**), divaricatic (**25**), perlatolic (**43**), protocetraric (**61**) and norstitic (**58**) acids and the also tested psoromic acid and lichexanthone. The compounds were evaluated against murine melanoma B16-F10, human melanoma UACC-62 and fibroblast cells NIH/3T3. The test was performed with sulforhodamine B (SRB) and the anticancer drug doxorubicin was used as positive control. The results from SRB assays were expressed as growth inhibition 50% rate (GI_50_) and lethal growth 50% (LC_50_), according to Holbeck [164] and also in terms of selectivity index (SI), when a value greater than three indicates that the neoplastic cells atr more sensitive to a certain compound than normal cells [133].

The test with SRB revealed a significant cytotoxic activity in UACC-62 cells with protocetraric acid (GI_50_ 0.52 µg∙mL^−1^ and SI 93.3), with an inhibitory concentration very close to the control doxorubicin (GI_50_ 0.47 µg∙mL^−1^ and SI 1.2), but with a significantly better selectivity index than the control. Other compounds such as divaricatic acid (GI_50_ 2.7 µg∙mL^−1^ andSI 5.4) and perlatolic acid (GI_50_ 3.3 µg∙mL^−1^ and SI 7.9) also had a good response to this cell line. Difractaic, usnic, norstitic and psoromic acids had intermediate sensitivity, with GI_50_ values ranging from 24.7–36.6 µg∙mL^−1^. Atranorin presented low sensitivity with a GI_50_ of 147.2 µg∙mL^−1^ and SI of 1.7 and lichexanthone was inactive against this lineage. For B16-F10 cells the best result was the divaricatic acid, showing high sensitivity and selectivity with GI_50_ 4.4 µg∙mL^−1^ and SI 3.3, followed by perlatolic, protocetraric, diffractaic acids with average sensitivity and usnic and nortictic acids with low sensitivity. Atranorin and lichexantone demonstrated no sensitivity to this lineage. For the 3T3 lineage, divaricatic acid showed the best result, with a GI_50_ of 14.5 µg∙mL^−1^. The remaining compounds presented GI_50_ values ranging from 26.0–248.6 µg∙mL^−1^. Atranorin and lichexantone also had no sensitivity against this lineage. The CL_50_ values only showed satisfactory results for the UACC-62 lineage for divaricatic (CL_50_ 19.5 µg∙mL^−1^) and perlatolic (CL_50_ 27.6 µg∙mL^−1^) acids as the other compounds showed values close to 250 µg∙mL^−1^, presenting low cytotoxicity, as with the other cell lineage where all the compounds demonstrated CL_50_ values close to or higher than 250 µg∙mL^−1^.

Singh *et al.* [157] studied the effect of usnic acid on the growth inhibition, cell induction cycle control and apoptosis in A549 human lung carcinoma cells using the MTT method. Treatment with usnic acid at the concentration of 25–100 µM for 24 and 48 h decreased the number of cells from 39%–67% and 68%–89%, respectively, and increased cell death two- to eight-fold, respectively. Usnic acid at the concentration of 1–10 µM also significantly suppressed the formation of A549 cell colonies. Inhibition of cell growth was associated with the control phase G0/G1. Usnic acid decreased the protein expression of cyclin dependent kinase CDK4, CDK6 and cyclin D1 and elevated the expression of inhibitory protein (CDK1) p21/cip1. When examined, cell death associated with molecular changes was observed whereby usnic acid induces mitochondrial membrane depolarization and leads to an increase in the cells apoptosis by more than twice. The effect of usnic acid on apoptosis was accompanied by increased poly(ADP-ribose)polymerase cleavage. The study thus showed that usnic acid inhibits cell growth involving the phase cell cycle G0/G_1_ control and induces cell death by the mitochondrial membrane depolarization and apoptosis of human lung carcinoma cells [157].

### 3.5. Anti-Inflammatory Activity

A galactofuranose heteropolysaccharide with predominant (1→5)-Gal*f* bonds and side chains in position 6, isolated from *Trebouxia* sp., a photobiont from *R. gracilis*, extracted by Cordeiro *et al.* [31] presented induction properties in the *in vitro* activation ofperitoneal macrophages at all tested concentrations (1–150 µg∙mL^−1^). At the concentration of 150 µg∙mL^−1^, there was a 60% increase of the macrophage activation compared to the control group, confirmed by scanning electron microscopy (SEM) [31].

A pharmaceutical composition containing ramalin with circulation anti-inflammatory effects was presented and patented by Yim *et al.* [11]. The action is manifested as a result of of iNOS expression suppression in the transcription stage and also by suppressing the creation of NO, which is a nuclear substance mediator in the inflammatory reactions, and the activation suppression of the nuclear transcription factor kappa B (NF-kB) which is a precursor of the inflammatory mediation by suppressing the signal transmission route from protein kinases p38 MAPK, ERK ½ and JNK and also suppressing the expression of Toll-like 4 (TLR4) which is a lipopolysaccharide receptor (LPS) [11].

Byeon *et al.* [138] presented data on the *in vitro* effects of stereocalpin-A (**138**) concerning the compound’s ability to suppress the expression of vascular cell adhesion molecules (VCAM-1), induced by TNF-α in vascular smooth muscle cells (VSMCs). The pretreatment of VSMCs for 2 hours with the substance at non-toxic concentrations of 0.1–10 µg∙mL^−1^ inhibited TNF-α, inducing the adhesion of monocytic THP-1 cells and the expression of vascular cell adhesion molecules (VCAM-1) and inner cell adhesion molecule (ICAM-1). The compound also reduced the phosphorylation of P38, ERK, JNK and Akt. Stereocalpin-A demonstrated anti-inflammatory activity due to the negative regulation of induced adhesion molecules by TNF-α and the expression of MCP-1, the adhesion of monocytes and production of reactive oxygen species (ROS) in vascular smooth muscle cells (VSMCs) exerting a protective effect by inflammation modulation inside the atherosclerotic lesion. Previous studies have mentioned the participation of VSCMs in the initiation of atherosclerosis [138].

Another invention developed by Yim *et al.* [139] presents a pharmaceutical composition containing stereocalpin-A which can inhibit the expression of cell adhesion molecules mediated by TNF-α, and therefore can be used to prevent or effectively treat arteriosclerosis [139].

### 3.6. Other Activities

Besides the biological activities presented above for the lichens from the genus *Ramalina*, other activitiesfound are presented in this section. Lichen metabolites collected in Mato Grosso do Sul State, Brazil, such as difractaic acid, atranorin, chloroatranorin, usnic acid and the artifact ethyl orsenilate were tested against the phytopathogenic fungus *Cladosporium sphaerospermum* using the bioautographic test by Honda *et al.* [165]. The results showed that these compounds effective inhibited the growth of the fungus [165]. 

Different species of Koreans and Chinese lichens were evaluated for their activity against the phytopathogenic fungus *Colletotrichum acutatum*, the causing agent of anthracnose in pepper, by Wei *et al.* [166]. Among the tested species, *R. conduplicans,* obtained 59.5% inhibition, the second highest rate of mycelial growth inhibition of the tested fungus, showing that lichens can be useful as new fungicidal natural sources [166].

The methanolic extracts from the lichens *Ramalina hossei* and *Ramalina conduplicans* had their anthelmintic efficacy assessed by Kumar *et al.* [94]. The results showed that both lichens exhibited anti-helminth activity in dose-dependent form, as revealed by paralysis and death of tested Indian adult worms [94].

The antimycobacterial activity of twenty-six compounds derived from lichens of four different families, included the family Ramalinaceae was tested. Most of the compounds already had been isolated from species of the genus *Ramalina*. The results showed that the diffractaic acid (**38**) was the most active compound, with MIC value 15.6 µg∙mL^−1^, followed by norstitic acid (**58**), 62.5 µg∙mL^−1^ and usnic acid (**17**) 62.5 µg∙mL^−1^. The hypostictic and protocetraric (**61**) acids showed moderate inhibitory activity, with MIC 94.0 µg∙mL^−1^ and 125 µg∙mL^−1^ respectively. The other compounds showed low inhibitory activity on the growth of *Mycobacterium tuberculosis*, with MIC values > 250 µg∙mL^−1^ [167].

Research by Lee *et al.* [8] showed that usimine C (**20**), from *R. terebrata*, induced proliferation of human dermal fibroblast cells CCD-986SK up to 1.6 times after treatment with 90 µg∙mL^−1^ during 48 h. The type I procollagen synthesis was significantly increased 1.3 times, 3 times and 5 times after treatment with 0.14, 0.72 and 3.6 µg of usimine-C/mL/24 h, respectively, while no significant increase was observed after treatment with usimine-A or -B [8]. The invention of a pharmaceutical composition containing usimine-C was presented and patented by Lim *et al.* [168] for proliferation of dermal epithelium fibroblast acting in collagen production, preventing, in this way, the formation of wrinkles [168].

The anti-schistosoma activity of the sulfated polysaccharide α-D-glucan (Glu.SO_4_) extracted from *R. celastri* were evaluated, after encapsulation in liposomes (Glu.SO_4_-LIPO), in mice infected with *Schistosoma mansoni*. The effect of treatment with Glu.SO_4_ and Glu.SO_4_-LIPO (10 mg∙kg^−1^) on the disposal of eggs, parasite load and liver granuloma formation was evaluated using Swiss albino female mice, between the ages of 35–40 days, weighing about 25 ± 2 g, infected with 150 cercariae/animal (*Biomphalaria glabrata*, BH strain). Four groups were studied containing 10 samples each (*N* = 10), two controls (empty liposomes and NaCl) and two treatment groups with Glu.SO_4_ and Glu.SO_4_-LIPO, using a single dose. The results of the parasitological analysis revealed that Glu.SO_4_-LIPO was as efficient as Glu.SO_4_ in the reduction of egg disposal and parasite load. Treatment with free Glu.SO_4_ and Glu.SO_4_-LIPO produced a statistically significant reduction in the number of granulomas, by 62% and 63%, respectively [169].

The larvicidal activity of some lichen metabolites like (+)-usnic acid, atranorin and gyrophoric acid found in species from the genus *Ramalina*, and also of 3-hydroxyphysodic acid, were tested against second and third stage larvae from the *Culiseta longiareolata* mosquito by Cetin *et al.* [170]. All metabolites presented high larvicidal properties. The LC_50_ values were 0.41 µg∙mL^−1^ for gyrophoric acid, 0.48 µg∙mL^−1^ for (+)-usnic acid, 0.52 µg∙mL^−1^ for atranorin and 0.97 µg∙mL^−1^ for 3-hydroxyphysodic acid. However, when LC_90_ values were compared, the best result was from the (+)-usnic acid with 1.54 µg∙mL^−1^, followed by gyrophoric acid with 1.93 µg∙mL^−1^, then 3-hydroxy-physodic acid with 4.33 µg∙mL^−1^ and finally the compound atranorin with 5.63 µg∙mL^−1^. Thus it was revealed that some lichen secondary metabolites have a promising role as larvicides [170].

Lim *et al.* [135] patented a pharmaceutical composition containing ramalin or one of its salts, that can be used as functional food for treating liver disease, which may inhibit liver fibrosis and reduce liver cirrhosis levels. When compared with silymarin (a protective drug in liver cells) in animal experiments significant results were achieved without liver cell cytotoxicity. In this way, the composition can be effectively used to prevent or treat liver fibrosis and liver cirrhosis [135].

Kosugi *et al.* [24] demonstrated that sugar arabitol, extracted from the green algae *Trebouxia* sp. lichen photobiont *R. yasudae* has the ability to increase the expression of drought-induced non-photochemical (NPQ-d), dissipating light energy excess and protecting the photobiont of photoinhibition [24].

## 4. Concluding Remarks

From the 246 lichens species that compose the genus *Ramalina* mentioned in the literature, about 47%, or about 118 species with studied chemistry and biological activity were described in this review, which covers a total of 153 isolated and identified compounds. As for biological activity, the percentage of species studied is even smaller, because if the total of 13 species is considered, only about 5% had some biological activity study. The vast majority of studies were with crude lichens extracts, and only 27 compounds, or about 18% of the 153 identified compounds, underwent biological tests.

However, the results are promising, besides the diversity of biological activities presented by crude extracts and the few compounds whose activities were tested, some of these activities have great importance for medicine, as is the case of the observed antitumor, cytotoxic, and anti-inflammatory properties, in some cases leading to patented pharmaceutical formulations containing lichen substances with medical purposes.

In this regard, due to the good results demonstrated by various extracts and some isolated compounds from the genus *Ramalina* that have shown promising potential, especially with antimicrobial, antioxidant, antitumor, cytotoxic, antiviral, anti-inflammatory properties, and for the prevention and treatment of liver disease, among other benefits, it can be concluded that other species of this genus that have been little or not studied deserve special attention with research involving both the chemical part and the biological one to increase the contribution to the discovery of new compounds that may serve as models for new drugs with therapeutic properties.

## Supplementary Materials

Supplementary materials can be accessed at: http://www.mdpi.com/1420-3049/20/05/8952/s1.
