# Supplementary material for: Chemistry and Biological Activity of Ramalina Lichenized Fungi
Source: Molecules. 2015 May 19;20(5):8952–87. doi: 10.3390/molecules20058952 (PMC6272487; doi:10.3390/molecules20058952)
Supplement: Supplementary file 1 [file molecules-20-08952-s001.pdf]

Supplementary Information

Table S1. Polysaccharides reported in the *Ramalina* species of lichenized fungi (Fungi: Ascomycota).

No.	Class of Compounds	Compounds Name	Source (*Ramalina species*)	References	Current Names Of Species *
**1**	Carbohydrates	α-Glucan	*R. celastri*	[1–6]	*R. celastri*
			R. dendriscoides	[7]	*R. dendriscoides*
			R. fraxinea	[7]	*R. fraxinea*
			R. gracilis	[7]	*R. gracilis*
			R. gracilis (photobiont)	[8]	*R. gracilis*
			R. peruviana	[7]	*R. peruviana*
			R. peruviana (mycobiont)	[9]	*R. peruviana*
			R. usnea	[10]	*R. australiensis*
**2**		Galactomannan	*R. celastri*	[3]	*R. celastri*
			R. dendriscoides	[7]	*R. dendriscoides*
			R. ecklonii	[1]	*R. ecklonii*
			R. fraxinea	[7]	*R. fraxinea*
			R. gracilis	[7]	*R. gracilis*
			R. gracilis (photobiont)	[8]	*R. gracilis*
			R. peruviana	[7]	*R. peruviana*
			R. peruvina (mycobiont)	[5]	*R. peruviana*
			R. usnea	[10,11]	*R. australiensis*
**3**		Glucose	*R. crassa* (mycobiont)	[12]	*R. siliquosa*
			R. sinensis	[13]	*R. sinensis*
			R. fraxinea	[14]	*R. fraxinea*
**4**		Galactose	*R. crassa* (phycobiont)	[12]	*R. siliquosa*
			R. fraxinea	[14]	*R. fraxinea*
**5**		Mannose	*R. sinensis*	[13]	*R. sinensis*
**6**		Rhamnose	*R. sinensis*	[13]	*R. sinensis*
			R. fraxinea	[14]	*R. fraxinea*
**7**		D-Arabitol	*R. reticulata*	[15]	*R. menziessi*
			R. siliquosa	[16]	*R. siliquosa*

No.	Class of Compounds	Compounds Name	Source (*Ramalina species*)	References	Current Names Of Species *
			R. calicaris	[17]	*R. calicaris*
			R. sinensis	[17]	*R. sinensis*
			R. tayloriana	[18]	*R. luciae*
			R. yasudae (photobiont)	[19]	*R. yasudae*
			R. geniculata	[20]	*R. inflata subsp. inflata*
			R. scopulorum	[20]	*R. siliquosa*
**8**		Mannitol	*R. fraxinea*	[14]	*R. fraxinea*
**9**		Glucosamine	*R. fraxinea*	[14]	*R. fraxinea*
**10**		Arabinose	*R. fraxinea*	[14]	*R. fraxinea*
**11**		Xylose	*R. fraxinea*	[14]	*R. fraxinea*
**12**		Glucoronic acid	*R. fraxinea*	[14]	*R. fraxinea*
**13**		Lentinan	*R. complanata*	[21]	*R. complanata*
**14**		Amylose	*R. celastri*	[3]	*R. celastri*
			R. celastri (photobiont)	[4]	*R. celastri*
			R. gracilis (photobiont)	[8]	*R. gracilis*
**15**		Galacturonic acid	*R. terebrata*	[22]	*R. terebrata*
**16**		Ribitol	*R. crassa* (phycobiont)	[23]	*R. siliquosa*
			R. subbreviuscula (phycobiont)	[23]	*R. subbreviuscula*

* Updating scientific names validated by Index Fungorum, available at http://www.indexfungorum.org/names/Names.asp in May 2014.

Table S2. Usnic acid and derivatives reported in the *Ramalina* species of lichenized fungi (Fungi: Ascomycota).

No.	Class of Compounds	Compounds Name	Source	References	Current Names of Species *
**17**	Usnic acid and derivatives	Usnic acid	*R. africana*	[24]	*R. africana*
			R. asahinae	[25]	*R. asahinae*
			R. boninensis	[26,27]	*R. boninensis*
			R. boulhautina	[28]	*R. boulhautiana*
			R. anceps	[29–31]	*R. anceps*
			R. atlantica	[32]	*R. cuspidata*
			R. breviuscula	[33]	*R. breviuscula*
			R. cactacearum	[34]	*R. cactacearum*
			R. calcarata	[30]	*R. calcarata*
			R. calicaris	[9,17,35,36]	*R. calicaris*
			R. camptospora	[29,30]	*R. camptospora*
			R. capitata	[36–38]	*R. capitata*
			R. celastri	[30,39–41]	*R. celastri*
			R. celastri (mycobiont)	[39]	*R. celastri*
			R. ceruchis	[42,34]	*R. ceruchis*
			R. ceruchoides	[42]	*R. ceruchoides*
			R. chilensis	[31,34,43,44]	*R. chilensis*
			R. cochlearis	[30]	*R. cochlearis*
			R. combeoides	[42]	*R. combeoides*
			R. complanata	[29,30]	*R. complanata*
			R. conduplicans	[45]	*R. conduplicans*
			R. crassa	[32,46]	*R. siliquosa*
			R. crassa (mycobiont)	[46,47]	*R. siliquosa*
			R. curnowii	[32,36]	*R. cuspidata*
			R. cuspidata	[9,48–50]	*R. cuspidata*
			R. cuspidata var. *armorica*	[51]	*R. cuspidata*
			R. cuspidata var. *cuspidata*	[51]	*R. cuspidata*

No.	Class of Compounds	Compounds Name	Source	References	Current Names of Species *
			R. cuspidate var. *stenoclada*	[51]	*R. cuspidata*
			R. darwiniana	[29]	*R. darwiniana*
			R. dendriscoides	[30]	*R. dendriscoides*
			R. dendroides	[30]	*R. dendroides*
			R. diracerata	[33,52]	*R. diracerata*
			R. druidarum	[32]	*R. siliquosa*
			R. ecklonii	[34,53]	*R. ecklonii*
			R. evernioides	[33]	*R. evernioides*
			R. farinacea	[9,24,33,43,50,54–63]	*R. farinacea*
			R. fastigiata	[37,36]	*R. fastigiata*
			R. flaccescens	[34,42]	*R. flaccescens*
			R. fragilis	[29]	*R. fragilis*
			R. fraxinea	[14,27,36–38,64]	*R. fraxinea*
			R. furcellangulida	[29]	*R. furcellangulida*
			R. geniculata	[65,66]	*R. inflate subsp. inflata*
			R. glaucescens	[67]	*R. glaucescens*
			R. gracilis	[30]	*R. gracilis*
			R. grumosa	[29,30]	*R. grumosa*
			R. hierrensis	[68]	*R. hierrensis*
			R. homalea	[42]	*Niebla homalea*
			R. hossei	[42]	*R. hossei*
			R. inanis	[34]	*R. inanis*
			R. inflata	[40]	*R. inflata*
			R. intermedia	[33]	*R. intermedia*
			R. kullensis	[69]	*R. kullensis*
			R. lacera	[43]	*R. lacera*
			R. landröensis	[50,69]	*R. landröensis*
			R. menziensii	[70]	*R. menziensii*
			R. minuscula	[50,69]	*R. dilacerata*

No.	Class of Compounds	Compounds Name	Source	References	Current Names of Species *
			R. montagnei	[29]	*R. montagnei*
			R. nervulosa	[71]	*R. nervulosa*
			R. obtusata	[27,33,50,69]	*R. obtusata*
			R. pacifica	[40,71]	*R. pacifica*
			R. paludosa	[72]	*R. paludosa*
			R. peranceps	[31,73]	*R. peranceps*
			R. peruviana	[30,34]	*R. peruviana*
			R. pollinaria	[27,37,59,66,74,75]	*R. pollinaria*
			R. polyforma	[29]	*R. polyforma*
			R. polymorpha	[36,37,59]	*R. polymorpha*
			R. prolifera	[30]	*R. prolifera*
			R. puiggarii	[30]	*R. puiggarii*
			R. pusilla	[30]	*R. inflate subsp. australis*
			R. rectangularis	[30]	*R. rectangularis*
			R. reticulata	[15,27,33,76,77]	*R. menziesii*
			R. rigida	[30]	*R. rigida*
			R. roesleri	[78]	*R. roesleri*
			R. scorpulorum	[16,49,50,69,79]	*R. siliquosa*
			R. sekika	[27]	*R. sekika*
			R. sharpii	[31]	*R. sharpii*
			R. sideriza	[29]	*R. sideriza*
			R. siliquosa	[9,16,32,47]	*R. siliquosa*
			R. siliquosa var. *crassa*	[51]	*R. siliquosa*
			R. siliquosa var. *siliquosa*	[51]	*R. siliquosa*
			R. siliquosa var. *x*	[51]	*R. siliquosa*
			R. siliquosa var. zopfii	[51]	*R. siliquosa*
			R. sinensis	[17,80]	*R. sinensis*
			R. sorediantha	[29]	*R. sorediantha*

No.	Class of Compounds	Compounds Name	Source	References	Current Names of Species *
			R. sorediosa	[29,30]	*R. sorediosa*
			R. stenoclada	[32]	*R. cuspidata*
			R. stenospora	[81]	*R. stenospora*
			R. subcomplanata	[43,82]	*R. subcomplanata*
			R. subfarinacea	[9,36,50,69]	*R. subfarinacea*
			R. subpollinaria	[30]	*R. subpollinaria*
			R. terebrata	[83–85]	*R. terebrata*
			R. tingitana	[86]	*R. tingitana*
			R. tayloriana	[18]	*R. luciae*
			R. thrausta	[50]	*R. thrausta*
			R. tumidula	[34]	*R. tumidula*
			R. usnea	[29–31,87]	*R. australiensis*
			R. yasudae	[43,65]	*R. yasudae*
			R. yasudae (mycobiont)	[46]	*R. yasudae*
**18**		Usimine A	*R. terebrata*	[83,85,88,89]	*R. terebrata*
**19**		Usimine B	*R. terebrata*	[83,85,88,89]	*R. terebrata*
**20**		Usimine C	*R. terebrata*	[83,85,88,89]	*R. terebrata*
**21**		*Iso*-usnic acid	*R. hierrensis*	[68]	*R. hierrensis*
**22**		Usninic acid	*R. bornholmiensis*	[50]	*R. bornholmiensis*
			R. calicaris	[35]	*R. calicaris*
			R. cuspidata	[50]	*R. cuspidata*
			R. farinacea	[50]	*R. farinacea*
			R. landroënsis	[50]	*R. landroënsis*
			R. minuscula	[50]	*R. dilacerata*
			R. obtusata	[50]	*R. obtusata*
			R. scopulorum	[50]	*R. siliquosa*
			R. subfarinacea	[50]	*R. subfarinacea*
			R. thrausta	[50]	*R. thrausta*

* Updating scientific names validated by Index Fungorum, available at http://www.indexfungorum.org/names/Names.asp in May 2014.

Table S3. Depsides reported in the *Ramalina* species of lichenized fungi (Fungi: Ascomycota).

No.	Class of Compounds	Compounds Name	Source (*Ramalina species*)	References	Current Names of Species *
**23**	Depsides	Sekikaic acid	*R. boulhautina*	[28,47]	*R. boulhautiana*
			R. calicaris	[17,27]	*R. calicaris*
			R. conduplicans	[45,90]	*R. conduplicans*
			R. chilensis	[31,44]	*R. chilensis*
			R. cochlearis	[30]	*R. cochlearis*
			R. darwiniana	[29]	*R. darwiniana*
			R. farinacea	[9,54,60,66,91]	*R. farinacea*
			R. farinacea var. nervulosa	[27]	*R. farinacea*
			R. fragilis	[29]	*R. fragilis*
			R. fraxinea	[14]	*R. fraxinea*
			R. furcellangulida	[29]	*R. furcellangulida*
			R. geniculata	[27,43,66]	*R. inflata subsp. inflata*
			R. glaucescens	[43,67]	*R. glaucescens*
			R. hossei	[45,92]	*R. hossei*
			R. luciae	[93]	*R. luciae*
			R. montagnei	[29]	*R. montagnei*
			R. nervulosa	[71,94]	*R. nervulosa*
			R. peruviana	[30,43,95]	*R. peruviana*
			R. polyforma	[29]	*R. polyforma*
			R. pusiola	[30]	*R. pusiola*
			R. roesleri	[78]	*R. roesleri*
			R. sekika	[27]	*R. sekika*
			R. sorediosa (race II)	[30]	*R. sorediosa*
			R. subcomplanata	[94]	*R. subcomplanata*
			R. subpollinaria (race I)	[30]	*R. subpollinaria*
			R. tayloriana	[18,43]	*R. luciae*
			R. usnea	[30,31,87]	*R. australiensis*

No.	Class of Compounds	Compounds Name	Source (*Ramalina species*)	References	Current Names of Species *
**24**		Atranorim	*R. celastri*	[30]	*R. celastri*
			R. combeoides	[42]	*R. combeoides*
			R. complanata	[30]	*R. complanata*
			R. darwiniana	[29]	*R. darwiniana*
			R. dendriscoides	[30]	*R. dendriscoides*
			R. ecklonii	[53]	*R. ecklonii*
			R. fragilis	[29]	*R. fragilis*
			R. furcellangulida	[29]	*R. furcellangulida*
			R. glaucescens	[67]	*R. glaucescens*
			R. hierrensis	[68]	*R. hierrensis*
			R. pacifica	[40]	*R. pacifica*
			R. paludosa	[72]	*R. paludosa*
			R. peruviana (mycobiont)	[95]	*R. peruviana*
			R. roesleri	[78]	*R. roesleri*
			R. siliquosa	[16,47]	*R. siliquosa*
			R. stenospora	[81]	*R. stenospora*
			R. subcomplanata	[82]	*R. subcomplanata*
			R. subpllinaria	[30]	*R. subpollinaria*
**25**		Divaricatic acid	*R. aspera* (race I)	[30]	*R. aspera*
			R. calcarata	[30]	*R. calcarata*
			R. complanata (race I)	[30]	*R. complanata*
			R. darwiniana	[29]	*R. darwiniana*
			R. furcellangulida	[29]	*R. furcellangulida*
			R. hierrensis	[68]	*R. hierrensis*
			R. homalea	[42]	*Niebla homalea*
			R. menziesii	[70]	*R. menziessi*
			R. polyforma	[29]	*R. polyforma*
			R. subbreviuscula	[96]	*R. subbreviuscula*
			R. usnea	[87]	*R. australiensis*

No.	Class of Compounds	Compounds Name	Source (*Ramalina species*)	References	Current Names of Species *
			R. usnea (race II)	[30]	*R. australiensis*
**26**		Homosekikaic acid	*R.cochlearis*	[30]	*R. cochlearis*
			R. conduplicans	[90]	*R. conduplicans*
			R. luciae	[93]	*R. luciae*
			R. nervulosa	[94]	*R. nervulosa*
			R. peruviana	[30,95]	*R. peruviana*
			R. pusiola	[30]	*R. pusiola*
			R. roesleri	[78]	*R. roesleri*
			R. sorediosa	[30]	*R. sorediosa*
			R. subcomplanata	[94]	*R. subcomplanata*
			R. subpollinaria	[30]	*R. subpollinaria*
			R. usnea	[30]	*R. australiensis*
**27**		Ramalinoic acid	*R. calicaris*	[27,47]	*R. calicaris*
			R. cochlearis	[30]	*R. cochlearis*
			R. farinacea	[27,43,97]	*R. farinacea*
			R. geniculata	[27]	*R. inflata subsp. Inflate*
			R. intermediella	[27,97]	*R. intermediella*
			R. nervulosa	[47]	*R. nervulosa*
			R. obtusata	[69]	*R. obtusata*
			R. peruviana	[43,95]	*R. peruviana*
			R. usnea	[87]	*R. australiensis*
			R. usneoides	[27]	*R. australiensis*
**28**		Obtusatic acid	*R. calicaris*	[9,35]	*R. calicaris*
			R. farinacea	[24,57]	*R. farinacea*
			R. fraxinea	[14]	*R. fraxinea*
			R. mediterranea	[98]	*R. mediterranea*
			R. obtusata	[27,47,52,69]	*R. obtusata*
			R. pollinaria	[27]	*R. pollinaria*
			R. sekika	[27]	*R. sekika*

No.	Class of Compounds	Compounds Name	Source (*Ramalina species*)	References	Current Names of Species *
**29**		Tumidulin	*R. ceruchis*	[34,99]	*R. ceruchis*
			R. chilensis	[34,44]	*R. chilensis*
			R. flaccescens	[34,99]	*R. flaccescens*
			R. inanis	[34]	*R. inanis*
			R. peruviana	[34]	*R. peruviana*
			R. tumidula	[34,100]	*R. tumidula*
**30**		4'-*O*-demethylsekikaic acid	*R. americana*	[47]	*R. Americana*
			R. cochlearis	[30]	*R. cochlearis*
			R. farinacea	[47]	*R. farinacea*
			R. peruviana	[30,95]	*R. peruviana*
			R. sorediosa	[30]	*R. sorediosa*
			R. usnea (races I and II)	[30]	*R. australiensis*
**31**		Evernic acid	*R. calicaris*	[9,35]	*R. calicaris*
			R. commixta	[27]	*R. commixta*
			R. farinacea	[24,57]	*R. farinacea*
			R. mediterranea	[98]	*R. mediterranea*
			R. pollinaria	[27,59,66,74,75]	*R. pollinaria*
			R. yasudae	[47,101]	*R. yasudae*
**32**		4'-*O*-methylnorhomosekikaic acid	*R. cochlearis*	[30]	*R. cochlearis*
			R. farinacea	[54]	*R. farinacea*
			R. peruviana	[30]	*R. peruviana*
			R. pusiola	[30]	*R. pusiola*
			R. subcomplanata	[94]	*R. subcomplanata*
			R. usnea (races I and II)	[30]	*R. australiensis*
**33**		4'-*O*-methylnorsekikaic acid	*R. cochlearis*	[30]	*R. cochlearis*
			R. farinacea	[47,54]	*R. farinacea*
			R. usnea (races I and II)	[30]	*R. australiensis*
**34**		2'-*O*-methylsekikaic acid	*R. asahinae*	[25,30,47,102]	*R. asahinae*

No.	Class of Compounds	Compounds Name	Source (*Ramalina species*)	References	Current Names of Species *
			R. grumosa	[30]	*R. grumosa*
			R. sorediosa	[30]	*R. sorediosa*
**35**		Chloroatranorin	*R. siliquosa*	[16]	*R. siliquosa*
			R. stenospora	[81]	*R. stenospora*
			R. subcomplanata	[82]	*R. complanata*
**36**		Chryptochorophaeic acid	*R. aspera* (race II)	[30]	*R. aspera*
			R. paludosa	[72,81]	*R. paludosa*
**37**		4’-*O*-demethylhomosekikaic acid	*R. peruviana*	[30]	*R. peruviana*
			R. usnea (races I and II)	[30]	*R. australiensis*
**38**		Diffractaic acid	*R. lacera*	[43]	*R. lacera*
			R. subcomplanata	[43,82]	*R. subcomplanata*
**39**		4-*O*-demethylbarbatic acid	*R. siliquosa* (mycobiont)	[103]	*R. siliquosa*
			R. siliquosa var. *Zopfii*	[47,51]	*R. siliquosa*
			R. subdecipiens	[47]	*R. subdecipiens*
**40**		Ramalinaic acid	*R. americana*	[47]	*R. americana*
			R. farinacea	[61]	*R. farinacea*
**41**		Gyrophoric acid	*R. americana*	[43]	*R. americana*
**42**		Trivaric acid	*R. americana*	[43,104]	*R. americana*
**43**		Perlatolic acid	*R. stenospora*	[81]	*R. stenospora*
**44**		4-*O*-demethylnorhomosekikaic acid	*R. peruvina*	[30]	*R. peruviana*
**45**		4’-*O*-methylsekikaic acid	*R. asahinae*	[30]	*R. asahinae*
**46**		4’-*O*-methylpaludosic acid	*R. asahinae*	[30,102]	*R. asahinae*
**47**		4,4’-di-*O*- methylcryptochlorophaeic acid	*R. asahinae*	[102]	*R. asahinae*
**48**		Boninic acid	*R. asahinae*	[25,26]	*R. asahinae*
**49**		Stenosporic acid	*R. stenospora*	[47,81]	*R. stenospora*
**50**		5-Hydroxysekikaic acid	*R. farinacea*	[91]	*R. farinacea*
**51**		5-Chlorosekikaic acid	*R. glaucescens*	[67]	*R. glaucescens*
**52**		Olivetoric acid	*R. leiodea*	[105]	*R. leiodea*

No.	Class of Compounds	Compounds Name	Source (*Ramalina species*)	References	Current Names of Species *
**53**		Paludosic acid	*R. paludosa*	[72]	*R. paludosa*
**54**		4-*O*-methyl-oxocryptochlorophaeic acid	*R. subfraxinea*	[106]	*R. subfraxinea*
**55**		Lecanoric acid	*R. lacera*	[43]	*R. lacera*
**56**		Bourgeanic acid	*R. bourgeana*	[107,108]	*R. bourgeana*

* Updating scientific names validated by Index Fungorum, available at http://www.indexfungorum.org/names/Names.asp in May 2014.

Table S4. Depsidones reported in the *Ramalina* species of lichenized fungi (Fungi: Ascomycota).

No.	Class of Compounds	Compounds Name	Source (*Ramalina species*)	References	Current Names of Species *
**57**	Depsidones	Salazinic acid	*R. anceps*	[31]	*R. anceps*
			R. angustissima	[27]	*R. subfarinacea*
			R. calcarata	[30]	*R. calcarata*
			R. chilensis	[31,43]	*R. chilensis*
			R. complanata	[29]	*R. complanata*
			R. complanata (races I and II)	[30]	*R. complanata*
			R. crassa	[32,47]	*R. siliquosa*
			R. crassa (mycobiont)	[46]	*R. siliquosa*
			R. darwiniana	[29]	*R. darwiniana*
			R. dendriscoides	[30]	*R. dendriscoides*
			R. farinacea	[43,54,109]	*R. farinacea*
			R. furcellangulida	[29]	*R. furcellangulida*
			R. nervulosa	[47]	*R. nervulosa*
			R. pacifica	[71]	*R. pacifica*
			R. peranceps	[31]	*R. peranceps*
			R. polyforma	[29]	*R. polyforma*
			R. rectangularis	[30]	*R. rectangularis*
			R. rigida	[30]	*R. rigida*

No.	Class of Compounds	Compounds Name	Source (*Ramalina species*)	References	Current Names of Species *
			R. scopulorum	[16,79]	*R. siliquosa*
			R. sharpie	[31]	*R. sharpie*
			R. siliquosa	[47]	*R. siliquosa*
			R. siliquosa var. *crassa*	[47,51]	*R. siliquosa*
			R. siliquosa (mycobiont)	[103]	*R. siliquosa*
			R. subbreviuscula	[96]	*R. subbreviuscula*
			R. subcomplanata	[94]	*R. subcomplanata*
			R. subfarinacea var. *reagens*	[58]	*R. subfarinacea*
			R. subfarinacea var. *salazinic*	[58]	*R. subfarinacea*
			R. sorediosa (races I, II and III)	[30]	*R. sorediosa*
			R. subfarinacea	[9,33,43,50,69]	*R. subfarinacea*
			R. subpollinaria (races I, II and III)	[30]	*R. subpollinaria*
			R. yasudae	[47,65]	*R. yasudae*
			R. yasudae (mycobiont)	[46]	*R. yasudae*
**58**		Norstictic acid	*R. anceps*	[30,31]	*R. anceps*
			R. angustissima	[27]	*R. subfarinacea*
			R. arabum	[43]	*R. arabum*
			R. chilensis	[31,43,44]	*R. chilensis*
			R. curnowii	[32]	*R. cuspidata*
			R. cuspidata var. *cuspidata*	[51]	*R. cuspidata*
			R. cuspidata var. *stenoclada*	[51]	*R. cuspidata*
			R. farinacea	[43,55,58,60,109]	*R. farinacea*
			R. gracilis (race II)	[30]	*R. gracilis*
			R. lacera	[43]	*R. lacera*
			R. peranceps	[47]	*R. peranceps*
			R. pusiola	[30]	*R. pusiola*
			R. sharpii	[31]	*R. sharpii*
			R. siliquosa	[9]	*R. siliquosa*
			R. stenoclada	[32]	*R. cuspidata*

No.	Class of Compounds	Compounds Name	Source (*Ramalina species*)	References	Current Names of Species *
			R. subfarinacea	[43]	*R. subfarinacea*
			R. subfarinacea var. *subfarinacea*	[110]	*R. subfarinacea*
			*R. subfarinac*ea var. *reagens*	[110]	*R. subfarinacea*
**59**		Hypoprotocetraric acid	*R. druidarum*	[32]	*R. siliquosa*
			R. cuspidata var. *armorica*	[51]	*R. cuspidata*
			R. cuspidata var. cuspidata	[51]	*R. cuspidata*
			R. farinacea	[109]	*R. farinacea*
			R. hypoprotocetraric	[47]	*R. farinacea*
			R. siliquosa	[16,111]	*R. siliquosa*
			R. siliquosa var. *druidarum*	[51]	*R. siliquosa*
			R. tumidula	[47]	*R. tumidula*
**60**		Scopuloric acid (or stictic)	*R. combeoides*	[42]	*R. combeoides*
			R. curnowii	[32]	*R. cuspidata*
			R. cuspidata var. *armorica*	[51]	*R. cuspidata*
			R. cuspidata var. *cuspidata*	[51]	*R. cuspidata*
			R. hierrensis	[68]	*R. hierrensis*
			R. scopulorum	[16,27,69,79]	*R. siliquosa*
**61**		Protocetraric acid	*R. farinacea*	[9,27,55,58,109]	*R. farinacea*
			R. lacera	[112]	*R. lacera*
			R. pacifica	[112]	*R. pacifica*
			R. siliquosa	[16,32]	*R. siliquosa*
			R. siliquosa (mycobiont)	[3]	*R. siliquosa*
			R. siliquosa var. *siliquosa*	[30,31]	*R. siliquosa*
**62**		Connorstictic acid	*R. anceps*	[30]	*R. anceps*
**63**		Criptostictic acid	*R. cuspidata* var. *armorica*	[51]	*R. cuspidata*
			R. cuspidata var. *cuspidata*	[51]	*R. cuspidata*
**64**		Peristictic acid	*R. cuspidata* var. *armorica*	[51]	*R. cuspidata*
**65**		Conhypoprotocetraric acid	*R. siliquosa* var. x	[51]	*R. siliquosa*
**66**		Variolaric acid	*R. hierrensis*	[68]	*R. hierrensis*

No.	Class of Compounds	Compounds Name	Source (*Ramalina species*)	References	Current Names of Species *
**67**		Gangaleodin	*R. hierrensis*	[68]	*R. hierrrensis*
**68**		Physodic acid	*R. leiodea*	[105]	*R. leiodea*
**69**		Coquimboic acid	*R. tumidula*	[113]	*R. tumidula*

* Updating scientific names validated by Index Fungorum, available at http://www.indexfungorum.org/names/Names.asp in May 2014.

Table S5. Fatty acids reported in the *Ramalina* species of lichenized fungi (Fungi: Ascomycota).

No.	Class of Compounds	Compounds Name	Source (*Ramalina species*)	References	Current Names of Species *
**70**	Fatty acids	Oleic acid	*R. lacera*	[43]	*R. lacera*
			R. celastri (mycobiont)	[41]	*R. celastri*
			R. yasudae	[114]	*R. yasudae*
**71**		Palmitic acid	*R. lacera*	[114]	*R. lacera*
			R. celastri (mycobiont)	[41]	*R. celastri*
**72**		Stearic acid	*R. lacera*	[43]	*R. lacera*
			R. celastri (mycobiont)	[41]	*R. celastri*
			R. yasudae	[114]	*R. yasudae*
**73**		α-Linolenic	*R. celastri* (mycobiont)	[43]	*R. celastri*
			R. yasudae	[114]	*R. yasudae*
**74**		Linoleic acid	*R. yasudae*	[114]	*R. yasudae*
**75**		Myristic acid	*R. yasudae*	[114]	*R. yasudae*
**76**		Arachidonic acid	*R. yasudae*	[114]	*R. yasudae*
**77**		D-Protolichesterinic acid	*R. almquistii*	[24,115]	*R. almquistii*
			R. roesleri	[78]	*R. roesleri*
**78**		Nephrosterinic acid	*R. almquistii*	[24,115]	*R. almquistii*

* Updating scientific names validated by Index Fungorum, available at http://www.indexfungorum.org/names/Names.asp in May 2014.

Table S6. Other compounds—Carotenoids reported in the *Ramalina* species of lichenized fungi (Fungi: Ascomycota).

No.	Class of Compounds	Compounds Name	Source (*Ramalina* species)	References	Current Names of Species *
**79**	Carotenoids	β-Cryptoxanthin	*R. ecklonii*	[116]	*R. ecklonii*
			R. usnea	[116]	*R. australiensis*
**80**		Lutein epoxide	*R. ecklonii*	[116]	*R. ecklonii*
			R. usnea	[116]	*R. australiensis*
**81**		Violaxanthin	*R. ecklonii*	[116]	*R. ecklonii*
**82**		Auroxanthin	*R. ecklonii*	[116]	*R. ecklonii*
			R. usnea	[116]	*R. australiensis*
**83**		Astaxanthin	*R. celastri*	[117]	*R. celastri*
			R. usnea	[116]	*R. australiensis*
**84**		Mutatoxanthin	*R. usnea*	[116]	*R. australiensis*
**85**		Lycoxanthin	*R. ecklonii*	[116]	*R. ecklonii*
**86**		Antheroxanthin	*R. ecklonii*	[116]	*R. ecklonii*
			R. usnea	[116]	*R. australiensis*
**87**		Ɛ-Caroten	*R. usnea*	[116]	*R. australiensis*
**88**		Zeaxanthin	*R. celastri*	[117]	*R. celastri*
**89**		β-Caroten	*R. celastri*	[117]	*R. celastri*
**90**		α-Doradexanthin	*R. celastri*	[117]	*R. celastri*
**91**		Lutein	*R. celastri*	[117]	*R. celastri*
			R. usnea	[116]	*R. australiensis*
**92**		Hydroxyechinenone	*R. ecklonii*	[116]	*R. ecklonii*
**93**		Diatoxanthin	*R. ecklonii*	[116]	*R. ecklonii*
**94**		Neoxanthin	*R. ecklonii*	[116]	*R. ecklonii*
			R. usnea	[116]	*R. australiensis*
**95**		Rhodoxanthin	*R. ecklonii*	[116]	*R. ecklonii*
			R. usnea	[116]	*R. australiensis*

* Updating scientific names validated by Index Fungorum, available at http://www.indexfungorum.org/names/Names.asp in May 2014.

Table S7. Other compounds—Steroids and terpenoids reported in the *Ramalina* species of lichenized fungi (Fungi: Ascomycota).

No.	Class of Compounds	Compounds Name	Source (*Ramalina* species)	Refences	Current Names of Species *
**96**	Steroids	α-Sitosterol	*R. africana*	[24]	*R. africana*
			R. hierrensis	[68]	*R. hierrrensis*
**97**		Brassicasterol	*R. africana*	[24]	*R. africana*
			R. tingitana	[86]	*R. tingitana*
**98**		Lichesterol	*R. africana*	[24]	*R. africana*
**99**		Ergosterol peroxide	*R. hierrensis*	[68]	*R. hierrensis*
			R. tingitana	[86]	*R. tingitana*
**100**		Cerevisterol	*R. hierrensis*	[68]	*R. hierrensis*
**101**	Terpenoids	Ursolic acid	*R. hierrensis*	[68]	*R. hierrensis*
**102**		*Iso*-arborinol acetate	*R. hierrensis*	[68]	*R. hierrensis*
**103**		Friedelin	*R. ecklonii*	[53]	*R. ecklonii*
**104**		(−)-Sandaracopimaric acid	*R. hierrensis*	[68]	*R. hierrensis*
**105**		Ceruchinol	*R. ceruchis* var. *tumidula*	[118]	*R. ceruchis*
			R. tigrina	[119]	*R. tigrina*

* Updating scientific names validated by Index Fungorum, available at http://www.indexfungorum.org/names/Names.asp in May 2014.

Table S8. Other compounds—Lipids and amines reported in the *Ramalina* species of lichenized fungi (Fungi: Ascomycota).

No.	Class of Compounds	Compounds Name	Source (*Ramalina* species)	References	Current Names of Species *
**106**	Lipids	Diacylglyceryl-*N,N,N*-trimethylhomoserine	*R. lacera*	[43]	*R. lacera*
**107**		Diacylglyceryltrimetylalanine	*R. lacera*	[43]	*R. lacera*
**108**		Phosphatidylcholine	*R. lacera*	[43]	*R. lacera*
**109**		Phosphatidyletanolamine	*R. lacera*	[43]	*R. lacera*
**110**		Phosphatidylinositol	*R. lacera*	[43]	*R. lacera*
**111**		Phosphatidic acid	*R. lacera*	[43]	*R. lacera*
**112**		Sulfoquinovosyldiacylglycerol	*R. lacera*	[43]	*R. lacera*
**113**		Monogalactosyldiacylglycerol	*R. celastri*	[120]	*R. celastri*
			R. lacera	[43]	*R. lacera*
**114**		Gigalactosyldiacylglycerol	*R. celastri*	[120]	*R. celastri*
			R. lacera	[43]	*R. lacera*
**115**		*O*-β-D-Galactopyranosyl-(1→1')-ceramide	*R. celastri*	[121]	*R. celastri*
**116**	Amines	Choline	*R. fraxinea*	[14]	*R. fraxinea*
**117**		Betaine	*R. fraxinea*	[14]	*R. fraxinea*
**118**		Histamine	*R. fraxinea*	[14]	*R. fraxinea*
**119**		Acetylcholine	*R. fraxinea*	[14]	*R. fraxinea*
**120**		β-Fenethylamine	*R. fraxinea*	[14]	*R. fraxinea*
**121**		Spermidine	*R. farinacea*	[122]	*R. farinacea*
**122**		Putrescine	*R. farinacea*	[123,124]	*R. farinacea*
**123**		Spermine	*R. calicaris*	[124]	*R. calicaris*

* Updating scientific names validated by Index Fungorum, available at http://www.indexfungorum.org/names/Names.asp in May 2014.

Table S9. Other compounds—Amino acids reported in the *Ramalina* species of lichenized fungi (Fungi: Ascomycota).

No.	Class of Compounds	Compounds Name	Source (*Ramalina* species)	References	Current Names of Species *
**124**	Amino acids	Glutamic acid	*R. celastri* (photobiont)	[4]	*R. celastri*
			R. fraxinea	[14]	*R. fraxinea*
			R. siliquosa	[125]	*R. siliquosa*
**125**		Aspartic acid	*R. celastri* (photobiont)	[4]	*R. celastri*
			R. fraxinea	[14]	*R. fraxinea*
			R. siliquosa	[125]	*R. siliquosa*
**126**		Alanine	*R. celastri* (photobiont)	[4]	*R. celastri*
			R. fraxinea	[14]	*R. fraxinea*
			R. siliquosa	[125]	*R. siliquosa*
			R. sinensis	[126]	*R. sinensis*
**127**		Serine	*R. fraxinea*	[14]	*R. fraxinea*
			R. siliquosa	[125]	*R. siliquosa*
**128**		Proline	*R. fraxinea*	[14]	*R. fraxinea*
			R. siliquosa	[125]	*R. siliquosa*
**129**		Arginine	*R. celastri* (photobiont)	[4]	*R. celastri*
			R. siliquosa	[125]	*R. siliquosa*
**130**		Glycine	*R. celastri* (photobiont)	[4]	*R. celastri*
			R. siliquosa	[125]	*R. siliquosa*
**131**		Lysine	*R. celastri* (photobiont)	[4]	*R. celastri*
			R. siliquosa	[125]	*R. siliquosa*
**132**		Leucine	*R. celastri* (photobiont)	[4]	*R. celastri*
			R. siliquosa	[125]	*R. siliquosa*
**133**		Threonine	*R. celastri* (photobiont)	[4]	*R. celastri*
			R. siliquosa	[125]	*R. siliquosa*
**134**		Glucosamine	*R. siliquosa*	[125]	*R. siliquosa*
**135**		γ-Aminobutiric acid	*R. fraxinea*	[14]	*R. fraxinea*
**136**		Taurine	*R. crassa*	[127]	*R. siliquosa*

* Updating scientific names validated by Index Fungorum, available at http://www.indexfungorum.org/names/Names.asp in May 2014.

Table S10. Other compounds reported in the *Ramalina* species of lichenized fungi (Fungi: Ascomycota).

No.	Class of Compounds	Compounds Name	Source (*Ramalina* species)	References	Current Names of Species *
**137**	Hidrazide	Ramalin	*R. terebrata*	[83,85,128–130]	*R. terebrata*
**138**	Cyclic peptide	Stereocalpin A	*R. terebrata*	[131–133]	*R. terebrata*
**139**	Phenolics compounds	2,3-Dihydroxy-4-methoxy-6-pentyl-phenylmethyl ester	*R. farinacea*	[91]	*R. farinacea*
**140**		Divaric acid	*R. africana*	[24]	*R. africana*
**141**		Ethyl divaricatinate	*R. africana*	[24]	*R. africana*
**142**		2-Hydroxy-4-methoxy-6-propyl benzoic acid	*R. roesleri*	[78]	*R. roesleri*
**143**		2,4-Dihydroxy-3,6-dimethyl-methyl ester benzoic acid	*R. roesleri*	[78]	*R. roesleri*
**144**		Isorhizonic acid	*R. dilacerata*	[134]	*R. dilacerate*
**145**	Organics acids and derivatives	α-Crotonic acid	*R. reticulata*	[15]	*R. menziesii*
**146**		Abscisic acid	*R. farinacea*	[123]	*R. farinacea*
**147**		Ethyl caprilate	*R. fastigiata*	[135]	*R. fastigiata*
**148**		Ethyl palmitate	*R. fastigiata*	[135]	*R. fastigiata*
**149**		Ethyl stearate	*R. fastigiata*	[135]	*R. fastigiata*
**150**	Benzopyran	Divaricat acid	*R. hierrensis*	[68]	*R. hierrensis*
**151**	Anthraquinone	Parientin	*R. hierrensis*	[68]	*R. hierrensis*
**152**	Hydrocarbon	Aspicilin	*R. ecklonii*	[34]	*R. ecklonii*
**153**		Ethylene	*R. lacera*	[47]	*R. lacera*

* Updating scientific names validated by Index Fungorum, available at http://www.indexfungorum.org/names/Names.asp in May 2014.

.

References

1. Miceno, A.M.; Gorin, P.A.J.; Iacomini, M. Galactomannan and isolichenan componentes of the carbohydrate-rich lichen *Ramalina ecklonii* (Spreng.) Mey. & Flot. *Agric. Biol. Chem.* **1991**, *55*, 1391–1392.
2. Teixeira, A.Z.A.; Iacomini, M.; Gorin, A.J. An unusual glucomannan from *Tornabenia intricata*. *Phytochemistry* **1992**, *31*, 3467–3470.
3. Stuelp, P.M.; Carneiro Leão, A.M.A.; Gorin, P.A.J.; Iacomini, M. The glucans of *Ramalina celastri*; relation with chemotypes of other lichens. *Carbohydr. Polym.* **1999**, *40*, 101–106.
4. Cordeiro, L.M.C.; Reis, R.A.; Tisher, C.A.; Gorin, P.A.J.; Ferreira, J.C.; Iacomini, M. Linear β-mannose containig polysaccharide, β-xylan, and amylose from the cultured photobiont Trebouxia sp. of the ascolichen *Ramalina celastri*. *FEMS Microbiol. Lett.* **2003**, *220*, 89–94.
5. Cordeiro, L.M.C.; Stocker-Wörgötter, E.; Gorin, P.A.J.; Iacomini, M. Elucidation of polysaccharide origin in *Ramalina peruviana* symbiosis. *FEMS Microbiol. Lett.* **2004**, *238*, 79–84.
6. Teixeira, A.Z.A.; Iacomini, M.; Gorin, P.A.J. Chemotypes of mannose-containing polysaccharides of lichens mycobionts: A possible aid in classification and identification. *Carbohydr. Res.* **1995**, *266*, 309–314.
7. Cordeiro, L.M.C.; Stocker-Wörgötter, E.; Gorin, P.A.J.; Iacomini, M. Comparative studies of the polysaccharides from species of the genus Ramalina-lichenized fungi-of three distinct habitats. *Phytochemistry* **2003**, *63*, 967–975.
8. Cordeiro, L.M.C.; Carbonero, E.R.; Sassaki, G.L.; Reis, R.A.; Stocker-Wörgötter, E.; Gorin, P.A.J.; Iacomini, M. A fungus-type β-galactofuranan in the cultivated *Trebouxia* photobiont of the lichen *Ramalina gracilis*. *FEMS Microbiol. Lett.* **2005**, *244*, 193–198.
9. Solberg, Y.J. Dyeing of wool with lichens and lichen substances. *Acta Chem. Scand.* **1956**, *10*, 1116–1123.
10. Gorin, P.A.J.; Iacomini, M. Polysaccharides of the lichens *Cetraria islandica* and *Ramalina usnea*. *Carbohydr. Res.* **1984**, *128*, 119–132.
11. Gorin, P.A.J.; Iacomini, M. Structural diversity of D-galacto-D-mannan components isolated from lichens having ascomycetous mycobionts. Carbohydr. *Res.* **1985**, *142*, 253–267.
12. Takahashi, K.; Takeda, T.; Shibata, S. Polysaccharides of lichen symbionts. *Chem. Pharm. Bull.* **1979**, *27*, 238–241.
13. Mamut, R.; Tursun, T.; Abbas, A. Analysis monosaccharide of the polysaccharide in *Ramalina sinensis* with HPLC. *Shipin Gongye* **2013**, *34*, 209–211.
14. Krzacek, T.; Smolarz, H. Chemical composition of *Ramalina fraxinea* (L.) Ach. Thallus. *Acta Soc. Bot. Pol.* **1979**, *48*, 27–34.
15. Stark, J.B.; Walter, E.D.; Owens, H.S. Method of isolation of usnic acid from *Ramalina reticulata*. *J. Am. Chem. Soc.* **1950**, *72*, 1819–1820.
16. Culberson, C.F. Some constituents of the lichen *Ramalina siliquosa*. *Phytochemistry* **1965**, *4*, 951–961.

17. Mittal, O.P.; Neelakantan, S.; Seshaadri, T.R. Chemical investigation of Indian lichens. XIV. Chemical components of *Ramalina calicaris* and *Ramalina sinensis*. *J. Sci. Ind. Res. India* **1952**, *11B*, 386–387.
18. Seshadri, T.R.; Subramanian, S.S. Indian lichens. VIII. Lichens growing on sandal trees. *Proc.-Indian Acad. Sci., Sect. A* **1949**, *30A*, 15–22.
19. Kosugi, M.; Miyake, H.; Yamakawa, H.; Shibata, Y.; Miyazawa, A.; Sugimura, T.; Satoh, K.; Itoh, S.; Kashino, Y. Arabitol provided by lichenous fungi enhances ability to dissipate excess light energy in a symbiotic green alga under desiccation. *Plant Cell Physiol.* **2013**, *54*, 1316–1325.
20. Ashina, Y.; Yanagita, M. Lichen substances. XXXIX. A new lichen acid, norstictinic acid, and the occurrence of D-arabitol in lichens. *Ber. Dtsch. Chem. Ges. B* **1934**, *67B*, 799–803.
21. Cordeiro, L.M.C.; Messias, D.; Sassaki, G.L.; Gorin, P.A.J.; Iacomini, M. Does apossymbiotically cultivated fungus *Ramalina* produce isolichenan? *FEMS Microbiol. Lett.* **2011**, *321*, 50–57.
22. McAvoy, K.; Igartuburu, J.M.; Romgni-Colvin, J. Polysaccharide composition of *Ramalina terebrata*. *Abstr. Pap. Am. Chem. Soc.* **2012**, *1*, 25–29.
23. Komiya, T.; Shibata, S. Polyols produced by the cultured phyco- and mycobionts of some *Ramalina* species. *Phytochemistry* **1971**, *10*, 695–699.
24. Shukla, V.; Negi, S.; Rawat, M.S.M.; Pant, G.; Nagatsu, A. Chemical study of *Ramalina africana* (Ramalinaceae) from the Garhwal Himalayas. *Biochem. Syst. Ecol.* **2004**, *32*, 449–453.
25. Islas, N.V.; Zambrano, A.; Rojas, J.L. Ozone Reactivity and Free Radical Scavenging Behavior of Phenolic Secondary Metabolites in Lichens Exposed to Chronic Oxidant Air Pollution from Mexico City. *J. Chem. Ecol.* **2007**, *33*, 1619–1634.
26. Asahina, Y.; Kusaka, T. Lichen substances. LXXXIII. A new depside, boninic acid; synthesis of boninic acid and of homosekikaic acid. *Ber. Dtsch. Chem. Ges. B* **1937**, *70B*, 1815–1821.
27. Asahina, Y.; Shibata, S. *Chemistry of Lichen Substances, A*; Asher & Co. Ltd: Vaals–Amsterdam, The Netherlands, 1971.
28. Huneck, S.; Troted, G. Lichen constituents. XXXIV. Structure and composition of *Ramalina boulhautiana*. *Z. Naturforschung B* **1966**, *21*, 904–904.
29. Aptroot, A.; Bungartz, F. The lichen genus *Ramalina* on the Galapagos. *Lichenologist* **2007**, *39*, 519–542.
30. Kashiwadani, H.; Kalb, K. The genus *Ramalina* in Brazil. *Lichenologist* **1993**, *25*, 1–31.
31. Rundel, P.W. Evolution relationships in the *Ramalina usnea* complex. *Lichenologist* **1978**, *10*, 141–156.
32. Culberson, W.L. Analysis of chemical and morphological variation in the *Ramalina siliquosa* species complex. *Brittonia* **1967**, *19*, 333–352.
33. Marshak, A.; Barry, G.T.; Craig, L.C. Antibiotic compound isolated from the lichen *Ramalina reticulate. Science* **1947**, *24*, 394–395.
34. Huneck, S.; Follmann, G. Lichen constituents. XXXVI. Chemistry of Chilean lichens. 15. Constituents of *Ramalina cactacearum*, var. ambigua, and *Medusulina chilena*. *Z. Naturforschung B* **1967**, *22*, 110–111.
35. Asahina, Y.; Fukuziro, F. Lichen substances.III. Constitution of obtusatic acid. *Ber. Dtsch. Chem. Ges. B* **1932**, *65B*, 580–583.

36. Wade, A.E. The Genus *Ramalina* in the British Isles. *Lichenologist* **1961**, *5*, 226–241.
37. Cansaran, D.; Atakol, O.; Halici, M.G.; Aksoy, A. HPLC analysis of usnic acid in some *Ramalina* species from Anatolia and investigation of their antimicrobial activities. *Pharm. Biol.* **2007**, *45*, 77–81.
38. Popiolek, Z. Content of lichen substances in some species of *Ramalina*. *Ann. Univ. Mariae Curie–Sklodowska Sect. C* **1967**, *22*, 165–168.
39. Fazio, A.T.; Adler, M.T.; Bertoni, M.D.; Sepúlveda, C.S.; Damonte, E.B.; Maier, M.S. Lichen secondary metabolites from the cultured lichen mycobionts of *Teloschistes chrysophthalmus* and *Ramalina celastri* and their antiviral activities. *Z. Naturforschung* C **2007**, *62*, 543–549.
40. Ramesh, P.; Baig, E.S.A. Chemical investigation of south Indian lichens: *Ramalina* species. *Indian Drugs* **1994**, *31*, 445–446.
41. Fazio, A.T.; Adler, M.; Maier, M.S. Usnic acid and triacylglycerides production by the cultured lichen mycobiont of *Ramalina celastri*. *Nat. Prod. Commun.* **2014**, *9*, 213–214.
42. Bendz, G.; Santesson, J.; Wachtmeister, C.A. Studies on the chemistry of lichens. *Acta Chem. Scand.* **1965**, *19*, 1185–1187.
43. Hanus, L.O.; Temina, M.; Dembitsky, V. Biodiversity of the chemical constituents in the epiphytic lichenized ascomycete *Ramalina* lacera grown on difference substrates *Crataegus sinaicus*, *Pinus halepensis*, and *Quercus calliprinos*. *Biomed. Pap.* **2008**, *152*, 203–208.
44. Huneck, S.; Troted, G. Lichen constituents. XX. Chemistry of Chilean lichens. 8. Componets of *Ramalina chilensis*. *Z. Naturforschung B* **1966**, *21*, 90–91.
45. Praven Kumar, S.V.; Kekuda, T.R.P.; Vinayaka, K.S.; Sudharshan, S.J. Anthelmintic and antioxidant efficacy of two macrolichens of Ramalinaceae. *Pharmacogn. J.* **2009**, *1*, 238–242.
46. Komiya, T.; Shibata, S. Formation of lichen substances by mycobionts of lichens. Isolation of (+)-usnic acid and salazinic acid from mybobionts of *Ramalina* sp. *Chem. Pharm. Bull.* **1969**, *17*, 1305–1306.
47. Huneck, S.; Yoshimura, I. *Identification of Lichen Substances*; Springer-Verlag: Berlin, Germany, 1996.
48. Bézivin, C.; Tomasi, S.; Lohézic-Le, F.; Boustie, J. Cytotoxic activity of some lichen extracts on murine and human cancer cell lines. *Phytomedicine* **2003**, *10*, 499–503.
49. Ryan, H.; O'Riordan, W.M. Tinctorial constituents of some lichens, which are used as dyes in Irland. *Proc. R. Irish Acad. B* **1917**, *33*, 91–104.
50. Salkowski, H. Circular polarization of usninic acids and other lichen substances. *Liebigs Ann. Chem.* **1911**, *377*, 123–126.
51. Parrot, D.; Jan, S.; Baert, N.; Guyot, S.; Tomasi, S. Comparative metabolite profiling and chemical study of *Ramalina siliquosa* complex using LC-ESI-MS/MS approach. *Phytochemistry* **2013**, *89*, 114–124.
52. Nakao, M. Chemical constituents of a Chinese drug "shi-hoa" and their constitutions. *Yakuga Zasshi* **1923**, *496*, 423–497.
53. Reyes, Q.A.; Vicuna, L.P.; Almonacid, V.J. Usnic acid and atranorin in some regional lichens. *Rev. Latinoam. Quim.* **1981**, *12*, 130–131.
54. Molho, L.; Bodo, B.; Molho, D. L'acide *O*-méthyl-4' norsékikaïque, nouveau metadepside isole d'um lichen du genre *Ramalina*. *Phytochemistry* **1979**, *18*, 2049–2051.

55. Tay, T.; Türk, A.Ö.; Yilmaz, M.; Türk, H.; Kivanç, M. Evaluation of the antimicrobial activity of the acetone extract of the lichen *Ramalina farinacea* and its (+)-usnic acid, norstitic acid, and protocetraric acid constituents. *Z. Naturforschung C* **2004**, *59*, 384–388.
56. Esimone, C.O.; Adikwu, M.U. Antimicrobial activity and cytotoxicity of *Ramalina farinacea*. *Fitoterapia* **1999**, *70*, 428–431.
57. Rao, P.S.; Shripathy, V. Chemical constituents of *Umbilicaria indica* Frey and *Ramalina farinacea* L. Ach. *Curr. Sci.* **1976**, *45*, 517–518.
58. Hawksworth, D.L. Typification of lichen farinaceous. *Bryologist* **1969**, *72*, 254–255.
59. Zopf, W. To the knowledge of the lichen material. *Liebigs Ann*. **1897**, *297*, 271–312.
60. Rangaswami, S.; Rao, V.S. Chemical components of *Ramalina farinacea*. *Indian J. Pharm.* **1954**, *16*, 197–199.
61. Hesse, O. Contribution for the knowledge of the lichens and their characteristic components. *J. Prakt. Chem.* **1903**, *68*, 1–71.
62. Koparal, A.T.; Tuylu, B.A.; Turk, H. In vitro cytotoxic activities of (+)-usnic acid and (−)-usnic acid on V79, A549, and human lymphocyte cells and their non-genotoxicity on human lymphocytes. *Nat. Prod. Res.* **2006**, *20*, 1300–1307.
63. Zopf, W. To the knowledge of the lichen substances. *Liebigs Ann. Chem.* **1905**, *340*, 276–309.
64. Briner, G.P.; Gream, G.E.; Riggs, N.V. Chemistry of Australian lichens. I. Some constituents of *Parmelia conspersa*, *Ramalina fraxinea*, *Usnea barbata*, and *Usnea ceratina* from the New England region. *Aust. J. Chem*. **1960**, *13*, 277–284.
65. Yamamoto, Y.; Mizuguchi, R.; Yamada, Y. Tissue cultures of *Usnea rubescens* and *Ramalina yasudae* and production of usnic acid in their cultures. *Agric. Biol. Chem.* **1985**, *49*, 3347–3348.
66. Asahina, Y.; Nonomura, S. Lichen substances. XVI. Constituents of *Ramalina* species with special reference to sekikaic acid. *Ber. Dtsch. Chem. Ges. B* **1933**, *66B*, 30–35.
67. Dias, D.A.; Urban, S. Phytochemical investigation of the Australian lichens *Ramalina glaucescens* and *Xanthoria parietina*. *Nat. Prod. Commun.* **2009**, *4*, 959–964.
68. Gonzalez, A.G.; Barrera, J.B.; Rodriguez, E.M.P.; Padron, C.E.H. Chemical constituents of the lichen *Ramalina hierrensis*. *Planta Med.* **1992**, *58*, 214–218.
69. Zopf, W. A contribution to the knowledge of the lichen constituents. *Liebigs Ann. Chem.* **1907**, *352*, 1–44.
70. Hauck, M.; Willenbruch, K.; Leuschner, C. Lichen substances prevent lichens from nutrient deficiency. *J. Chem. Ecol.* **2009**, *35*, 71–73.
71. Verma, N.; Behera, B.C.; Joshi, A. Studies on nutritional requirement for the culture of lichen *Ramalina nervulosa* and *Ramalina pacifica* to enhance the production of antioxidant metabolites. *Folia Microbiol.* **2012**, *57*, 107–114.
72. Culberson C.F. Chemical constituents of *Ramalina paludosa*. *Bryologist* **1967**, *70*, 397–405.
73. Urdapilleta, A.A.A. Atividade de Substâncias Isoladas de Líquens Sobre Formas Promastigotas de *Leishmania* (L.) *amazonensis* e *Leishmania* (V.) *braziliensis*. Master's Thesis, Universidade de Brasília, Brasilia-DF, 3 March 2006.
74. Morozova, O.V.; Dargaeva, T.D.; Brutko, L.I. Components of *Ramalina pollinaria*. *Khim. Prirod. Soed.* **1984**, *20*, 252–253.

75. Koller, G. Ramalic acid. *Monatsh. Chem.* **1932**, *61*, 286–292.
76. Kirk, P.M.; Cannon, P.F.; Minter, D.W.; Stalpers, J.A. *Dictionary of the Fungi*; CABI—Europe-UK, Cromwell Press: Trowbridge, UK, 2008.
77. Marshak, A.G. Extracting Usnic Acid. US 2557987 A, 26 June 1951.
78. Sisodia, R.; Geol, M.; Verma, S.; Rani, A.; Dureja, P. Antibacterial and antioxidante activity of lichen species *Ramalina roesleri*. *Nat. Prod. Res.* **2013**, *27*, 2235–2239.
79. Curd, F.H.; Robertson, A. Lichen acids. Part VI. Constituensts of *Ramalina scopulorum*. *J. Chem. Soc.* **1935**, *329*, 1379–1381.
80. Tugunay, T.; Reyim, M.; Adilijiang, A.A. Content determination and antioxidant activity evaluation of usnic acid in *Ramalina sinensis* Jatta. *Shipin Kexue* **2010**, *31*, 150–152.
81. Culberson, C.F. Stenosporic acid, a new depside in *Ramalina stenospora*. *Phytochemistry* **1970**, *9*, 841–844.
82. Kumar, K.C.S.; Banskota, A.H.; Manandhar, M.D. Chemical constituents and antibacterial activity of *Ramalina subcomplanata*. *J. Nepal Chem. Soc.* **1995**, *14*, 28–35.
83. Yim, J.H.; Lee, H.K.; Bhattarai, H.D.; Babita, P.; Kim, I.C.; Hong, S.G.; Kim, D.K.; Lee, Y.K.; Gu, S.; Oh, H.C. Antioxidant Activities of Ramalin Isolated from *Ramalina terebrata* Extracts. WO 2010053327 A2, 14 May 2010.
84. Huneck, S.; Follmann, G. The presence of usnic acid in *Lecanora melanophthalma* and *Ramalina terebrata*. *Naturwissenschaften* **1964**, *51*, 291–292.
85. Paudel, B.; Bhattarai, H.D.; Lee, H.K.; Oh, H.; Shin, H.W.; Han, J.; Yim, J.H. Antibacterial activities of ramalin, usnic acid and its tree derivatives isolated from the Antarctic lichen *Ramalina terebrata. Z. Naturforschung C* **2010**, *65*, 34–38.
86. Gonzalez, A.G.; Martin, J.D.; Martin, M.M. Chemical components of lichens. II. *Ramalina tingitana*. *Anal. Quim.* **1973**, *69*, 807–809.
87. Imshaug, H.A. Typification of *Ramalina usnea* (L.) R. H. Howe. *Lichenologist* **1972**, *5*, 317–318.
88. Lee, S.G.; Koh, H.Y.; Oh, H.; Han, S.J.; Kim, I.C.; Lee, H.K.; Yim, J.H. Human dermal fibroblast proliferation activity of usimine-C from Antarctic lichen *Ramalina terebrata. Biotechnol. Lett.* **2010**, *32*, 471–475.
89. Yim, J.H.; Kim, I.C.; Lee, S.G.; Kim, D.K.; Han, S.J.; Lee, H.S.; Seok, K.H.; Kim, S.J.; Kim, T.K.; Kang, P.S.; *et al.* Pharmaceutical Composition for the Treatment of Inflammatory Disease or Immune Disease Containing Ramalin. WO 2011152671 A2, 8 December 2011.
90. Luo, H.; Wei, X.; Yamamoto, Y.; Liu, Y.; Wang, L.; Jung, J.S.; Koh, Y.J.; Hur, J.-S. Antioxidant activities of edible lichen *Ramalina conduplicans* and its free radical scavenging constituents. *Mycoscience* **2010**, *51*, 391–395.
91. Lai, D.; Odimewu, D.; Esimone, C.; Grunwald, T.; Proksch, P. Phenolic compounds with *in vitro* activity against respiratory syncytial from the Nigerian lichen *Ramalina farinacea*. *Planta Med.* **2013**, *79*, 1440–1446.
92. Vinayaka, K.S.; Kekuda, T.R.P.; Swathi, D.; Praveen Kumar, S.V. Studies on chemical composition and *in vitro* antibacterial activity of solvent extracts of the lichen *Ramalina hossei* Vain. *BioTechnol. Indian J.* **2009**, *3*, 309–311.
93. Molho, D.; Bodo, B.; Culberson, W.L. A chemically distinctive new *Ramalina* from Fiji. *Bryologist* **1981**, *84*, 396–398.

94. Hamada, N. Individual variation in the contents of lichen substances in *Ramalina nervulosa* and *Ramalina subcomplanata*. *Bryologist* **1988**, *91*, 95–97.
95. Cordeiro, L.M.C.; Iacomini, M.; Stocker-Wörgötter, E. Culture studies and secondary compounds of six *Ramalina* species. *Mycol. Res.* **2004**, *108*, 489–497.
96. Hamada, N. The effect of temperature on lichen substances in *Ramalina subbreviuscula* (Lichens). *Bot. Mag.* **1983**, *96*, 121–126.
97. Asahina, Y.; Kusaka, T. Lichen substances. LXV. A new depside, ramalinolic acid. *Ber. Dtsch. Chem. Ges. B* **1936**, *69B*, 450–455.
98. Huneck, S. Lichen constituents. LXXXIX. Secondary substances of some lichens. *Phytochemistry* **1972**, *11*, 1493–1495.
99. Bendz, G.; Santesson, J.; Wachtmeister, C.A. Chemistry of lichens. XXI. Isolation and synthesis of methyl 3,5-dichlorolecanorate, a new depside from *Ramalina* spp. *Acta Chem. Scand.* **1965**, *19*, 1188–1190.
100. Huneck, S. Lichen constituents. XXXIV. Structure of tumidulin, a new chlorine containing depside. *Chem. Ber.* **1966**, *99*, 1106–1110.
101. Ingólfsdóttir, K. Usnic acid. *Phytochemistry* **2002**, *61*, 729–736.
102. Chester, D.O.; Elix, J.A. The identification of four new meta-depsides in the lichen *Ramalina asahinae*. *Aust. J. Chem.* **1978**, *31*, 2745–2749.
103. Hamada, N.; Ueno, T. Depside from an isolated lichen mycobiont. *Agric. Biol. Chem.* **1987**, *51*, 1705–1706.
104. Culberson, C.F.; LaGreca, S.; Johnson, A.; Culberson, W.L. Trivaric acid, a new tridepside in the *Ramalina americana* chemotype complex (lichenized Ascomycota: Ramalinaceae). *Bryologist* **2000**, *102*, 595–601.
105. Elix, J.A.; Peculiarities of the Australiasian lichen flora: Accessory metabolites, chemical and hybrid strains. *J. Hattori Bot. Lab.* **1982**, *52*, 407–415.
106. Culberson, C.F.; Culberson, W.L.; Johnson, A. A novel meta-depside from the *Ramalina subfraxinea* complex (Ascomycotina, Ramalinaceae). *Bryologist* **1990**, *93* 193–196.
107. Bodo, B.; Hebrard, P.; Molho; L.; Molho, D. Desmaziera evernioides and *Ramalina bourgeana*. *Tetraedron Lett.* **1973**, *18*, 1631–1634.
108. Bodo, B. Bourgeanic acid: A new metabolite of lichens. Structure, synthesis and biosynthesis. *Bull. Mus. Natl. Hist. Nat. Sci. Phys.-Chim.* **1975**, *3*, 23–97.
109. Stocker-Worgotter E.; Elix, J.A.; Grube, M. Secondary chemistry of lichen forming fungi: Chemosyndromic variation and DNA analyses of cultures and chemotypes in the *Ramalina farinacea*. *Bryologist* **2004**, *107*, 152–162.
110. Hawksworth, D.L. Chemical strains of the lichen *Ramalina subfariancacea*. *Bot. Notiser* **1968**, *121*, 317–320.
111. Culberson, C.F. Microchemical tests for the lichen depsidone hypoprotocetraric acid. *Bryologist* **1965**, *68*, 301–304.
112. Stuelp-Campelo, P.Ma.; de Oliveira, M.B.M.; Leão, A.M.A.C.; Carbonero, E.R.; Gorin, P.A.J.; Iacomini, M. Effect of a soluble α-D-glucan from the lichenized fungus *Ramalina celastri* on macrophage activity. *Int. Immunopharmacol.* **2002**, *2*, 691–698.

113. Huneck, S.; Lehn, J.M. Lichen constituents. XXVII. Identity between coquimboic acid and hypoprotocetraric acid. *Z. NaturforschungB* **1966**, *21*, 299–299.
114. Yamamoto Y.; Watanabe, A. Fatty acid composition of lichens and their phyco and mycobionts. *J. Gen. Appl. Microbiol.* **1974**, *20*, 83–86.
115. Hirayama, T.; Fujikawa, F.; Kasahara, T.; Otsuka, M.; Nishida, N.; Mizuno, D. Anti-tumor activities of some lichen products and their degradation products. *Yakugaku Zasshi* **1980**, *100*, 755–759.
116. Czeczuga, B.; de Corona, L.F. Carotenoids in lichens from Argentina. *Biochem. Syst. Ecol.* **1987**, *15*, 519–522.
117. Dembitsky, V.M. Lipids of lichens. *Prog. Lipid Res.* **1992**, *31*, 373–397.
118. Lehn, J.M.; Huneck, S. Lichen constituents. XVIII. First separation of the diterpene (−)-16-α-hydroxykaurane from a lichen. *Z. Naturforschung B* **1965**, *20*, 1013-1013.
119. Huneck, S.; Follmann, G. Lichen constituents. XXVIII. Chemistry of Chilean lichen. Compounds from *Ramalina tigrina*, and *Ramalina inanis*. *Z. Naturforschung B* **1966**, *21*, 713–714.
120. Sassaki, G.L.; Cruz, L.M.; Gorin, P.A.J.; Iacomini, M. Fatty acid composition of lipids present in selected lichenized fungi: A chemotyping study. *Lipids* **2001**, *36*, 167–174.
121. Machado, M.J.; Guerrini, M.; Gorin, P.A.J.; Torri, G.; Iacomoni, M. A galactosphingolipid from the lichen, *Ramalina celastri*. *Phytochemistry* **1997**, *45*, 651–653.
122. Cetin, H.; Tufan-Cetin, O.; Turk, A.O.; Tay, T.; Candan, M.; Yanikoglu, A.; Sumbul, H. Insecticidal activity of major lichen compounds, (−)- and (+)-usnic acid, against the larvae of house mosquito, *Culex pipiens* L. *Parasitol. Res.* **2008**, *102*, 1277–1279.
123. Unal, D.; Senkardesler, A. Abscisic acid and polyamine contents in the lichens *Pseudevernia furfuracea* and *Ramalina farinacea*. A. Sukatar, *Russ. J. Plant Physiol.* **2008**, *55*, 115–118.
124. Mateos, J.L.; Legaz, M.E. Presence and Identification of Polyamines and their Conjugation to Phenolics in Some Epiphytic Lichens. *Eukaryotism and Symbiosis*; Springer-Verlag: Berlin, Germany, 1997; pp. 491–498.
125. Solberg, Y.J. Studies on the chemistry of lichens. IX Quantitative determination of monosaccharides and amino acids in hydrolysates of several Norwegian lichen species. *Lichenologist* **1970**, *4*, 283–288.
126. Ramakrishnan, S.; Subrmanian, S.S. Amino acids of Lobaria subisidiosa, Umbilicaria pustulata, Parmelia nepalensis, and Ramalina sinensis. *Curr. Sci. Indian* **1966**, 35, 124–125.
127. Fujikawa, F.; Hirai, K.; Hirayama, T.; Toyota, T.; Urasaki, M.; Takagawa, M.; Fukuda, M.; Moritani, K.; Sanae, T. Free amino acids in lichens of Japan. *Yakugaku Zasshi* **1972**, *92*, 823–827.
128. Paudel, B.; Bhattarai, H.D.; Koh, H.Y.; Lee, S.G.; Han, S.J.; Lee, H.K.; Oh, H.; Shin, H.W.; Yim, J.H. Ramalin, a novel nontoxic antioxidant compound from the Antarctic lichen *Ramalina terebrata*. *Phytomedicine* **2011**, *18*, 1285–1290.
129. Yim, J.H.; Kim, I.C.; Han, S.J.; Lee, H.S.; Battarai, H.D.; Yang, S-G.; Lee, D.H. Ramalin for Preveting or Treating Hepatic Fibrosis and Liver Cirrhosis. WO 2013129714 A1, 6 September 2013.
130. Yim, J.H.; Kim, I.C.; Lee, S.G.; Kim, D.G.; Han, S.J.; Lee, H.S.; Kim, S.J.; Kim, T.G.; Kang, P.S.; Park, H.Y. Ramalin for Preveting and Treating Inflammatory Diseases and Immune Diseases. KR 1290745 B1, 29 September 2013.

131. Seo, C.; Yim, J.H.; Lee, H.K.; Park, S.M.; Sohn, J.H.; Oh, H. Stereocalpin A, a bioactive cyclic depsipeptide from the Antarctic lichen *Stereocaulon alpinum*. *Tetrahedron Lett.* **2008**, *49*, 29–31.
132. Byeon, H.E.; Park, B.K.; Yim, J.H.; Lee, H.K.; Moon, E.Y.; Rhee, D.K.; Pyo, S. Stereocalpin A inhibits the expression of adhesion molecules in activated vascular smooth muscle cells. *Int. Immunopharmacol*. **2012**, *12*, 315–325.
133. Yim, J.H.; Kim, I.C.; Kim, D.G.; Han, S.J.; Lee, H.S.; Oh, H.C.; Pyo, S.N. Stereoclapin-A for preveting or treating arteriosclerosis. KR 20130043995 A, 02 May 2013.
134. Pfau, A.S. Constituents of lichens. II. Constitution of barbatinic acid. *Helv. Chim Acta* **1928**, *11*, 864–876.
135. Huang, W.; Dai, W. The constituents of absolute from *Ramalina fastigiata* (Pors) Ach. The main ethyl ester of fatty acids. *Nat. Prod. Res. Dev.* **1989**, *1*, 27–28.
